# Four months vitamin D supplementation to vitamin D insufficient individuals does not improve muscular strength: A randomized controlled trial

**DOI:** 10.1371/journal.pone.0225600

**Published:** 2019-12-16

**Authors:** Guri Grimnes, Julia Kubiak, Rolf Jorde

**Affiliations:** 1 Tromsø Endocrine Research Group, Department of Clinical Medicine, UiT The Arctic University of Norway, Tromsø, Norway; 2 Division of Internal Medicine, University Hospital of North Norway, Tromsø, Norway; University of Newcastle, AUSTRALIA

## Abstract

**Main objective:**

The inconsistent results on the effects of vitamin D on muscle strength reported by intervention trials may partly be explained by inclusion of vitamin D sufficient individuals. The main objective was to study whether vitamin D supplementation will improve muscle strength in men and women with low serum vitamin D status, as measured by 25-hydroxyvitamin D (25(OH)D) at baseline.

**Methods:**

417 men and women aged 40–80 years were included and randomized to receive a loading dose of 100 000 IU (2500 ug) vitamin D_3_ followed by 20 000 IU (500 ug)/week, or placebo. Muscle strength was tested by dynamometers at baseline and after four months.

**Results:**

Serum 25(OH)D levels increased from 32.6±11.1 nmol/l to 88.8±19.4 nmol/l (p<0.01) in the vitamin D group, while remaining low in the placebo group (baseline and final levels at 35.1±13.6 nmol/l and 30.7 ±9.7 nmol/l respectively). Muscle strength (hip flexion, biceps flexion, pectorals and handgrip strength) did not change in any of the groups. The results were the same in analyses stratified on sex, 25(OH)D above/below 25 nmol/L (10 ng/ml); smoking status; and BMI above/below 27 kg/m^2^.

**Conclusion:**

These data does not support vitamin D supplementation for improving muscle strength.

## Introduction

Vitamin D deficiency is common [1], and associated with numerous negative health outcomes [2]. In particular, vitamin D is important for skeletal health, as deficiency may lead to rickets or osteomalacia [3]. The effects of vitamin D on fractures and falls have recently been questioned [4].

Muscle weakness and pain are commonly seen in osteomalacia [5], vitamin D receptors are present in muscle cells [6,7], and several observational studies have reported inverse associations between serum 25-hydroxyvitamin D (25(OH)D) levels and muscle strength [8,9]. Possible mechanisms include changes in muscle morphology (with atrophy of type 2 muscle fibers [10] as well as increased fatty infiltration in skeletal muscle [11]), improved mitochondrial function with vitamin D supplementation [12], and by optimizing contractility through regulation of calcium and phosphate handling [13]. Taking these results into account, one could expect an effect on muscle strength and function with vitamin D supplementation, but as the results from randomized controlled trials (RCTs) have been inconsistent, meta-analyses have concluded differently [14,15]. Thus, Beaudart et al. reported in 2014 in a systematic review and meta-analysis of RCTs using vitamin D a small, but significant effect of vitamin D supplementation on global muscle strength, which was more evident in people with 25(OH)D < 30 nmol/l as well as in people ≥ 65 years of age [14]. On the other hand, no effect was found in a similar systematic review and meta-analyses by Rosenahl-Riise et al. in 2017 [15].

Some of the heterogeneity of the results may be a reflection of different study designs, where a common pitfall is to include participants with sufficient vitamin D status (measured as 25-hydroxyvitamin D (25(OH)D)) at baseline [16,17]. Another limitation of the available studies, is that most of them are performed only with women [15].

We had the opportunity to include healthy community-dwelling participants of both genders and with low basal serum 25(OH)D levels from a large population-based study, in a randomized controlled trial with vitamin D supplementation over four months, assessing changes in standardized measurements of muscle strength. We hypothesized that vitamin D supplementation to vitamin D insufficient persons would improve muscle strength as compared to placebo.

## Material and methods

### Subjects

The Tromsø study is a population based health survey that was first conducted in 1974 [18]. In the seventh wave in 2015/2016, all citizens aged 40 years and older (n = 32 591) living in the municipality of Tromsø in northern Norway were invited to participate, where 21 083 attended. Serum 25(OH)D was measured on an ongoing basis in a total of 20922 participants, and based on the distribution, those with levels below the 7th percentile (<42 nmol/L (16.8 ng/ml)) and age <80 years were invited to participate in this study. Of 1489 subjects invited, 639 responded positively and were phone screened by the study nurses from the Clinical Research Unit at the University Hospital of North Norway. A standardized medical interview was performed in order to exclude subjects with known granulomatous disease, renal stones last five years, or serious diseases apparent through interview or screening blood tests that would make the subject unfit for participation. Patients with diabetes were excluded from participation due to one of the primary outcomes insulin resistance. Additionally, subjects with use of vitamin D supplements exceeding 800 IU (20 ug) vitamin D per day, subjects who used solarium on a regular basis, or were planning holiday(s) in tropical areas during the study period were excluded from participation. Women of childbearing potential (below the age of 50 years) without use of contraception were excluded. Weight and height and smoking status were asked for and used in the randomization procedure.

### Study design

The main end point of the study was effects on cardio-vascular risk factors, and the study design has been described in detail elsewhere [19]. In short, those who fulfilled the inclusion criteria, came to the first visit at the Clinical Research Unit of the University Hospital of North Norway for signing of the informed consent form, clinical examinations, height and weight measurements wearing light clothing and no shoes, medical history and fasting blood samples which included hemoglobin, liver enzymes, calcium, creatinine, thyroid hormones and TSH, high-sensitive CRP, sedimentation rate and glycated hemoglobin. If these examinations did not reveal any contraindication for participation, the next visit was performed within 2–5 days. At this second visit, the muscle strength testing was performed and the study drugs (20 000 IU (500 ug) cholecalciferol capsules Dekristol, Mibe, Jena, Germany) or identical looking placebo capsules containing arachis oil (Hasco-Lek, Wroclaw, Poland) were dispensed. Five capsules were given as a loading dose, followed by one capsule each week. The subjects were asked not to take any vitamin D supplements (including cod liver oil) during the four months intervention period.

The randomization was stratified according to gender, vitamin D status in the Tromsø study (serum 25(OH)D above/below 25 nmol/L (10 ng/ml)), smoking status (current smoking or not) and BMI above/below 27 kg/m^2^ (self-reported height and weight). Based on this, the randomization unit assigned the subject a randomization number using a block randomization procedure with random block sizes of 2, 4 or 6. This number was sent to the Clinical Research Unit (who did not have the randomization key) and to the hospital’s pharmacy (who had the key), with the latter dispensing the medication accordingly. Except for the pharmacy, which had no contact with the study participants, all nurses, doctors, other study personnel and study participants were blinded for the randomization throughout the study.

Two months after inclusion, a study nurse contacted the participants by phone to ask for adverse events and remind them to take the study medication. At four months the third visit was performed with examinations identical to the first visit. The fourth visit followed a few days later, with return of study medication and examinations identical to the second visit. Compliance was calculated as the ratio between capsules used (capsules supplied minus capsules returned) and number of weeks between second and fourth visit.

### Muscle strength testing

Handgrip strength was measured using a Jamar Plus dynamometer (Jamar Technologies, Lakewood, NJ, USA). The dominant side was used, but if there was a known injury or functional loss in the dominant side, the non-dominant side was preferred, also for the repeated final examination. Hip flexion strength, pectoralis strength and biceps strength were measured by use of the hand-held dynamometer Jtech Commander Muscle Tester (Jtech Medical Industries, Midvale, Utah, USA). Use of hand-held dynamometry have been shown to be a reliable and valid instrument for muscle strength testing as compared to the gold standard isokinetic dynamometry [20]. Standardized procedures were followed, with detailed description of how to place and stabilize the patient in order to isolate the muscle group being tested. External belt-fixation was used for hip and elbow flexion in order to avoid impact from the strength of the research nurse [21]. The participants were carefully instructed how to perform the tests. The participant was asked to press maximally towards the fixed dynamometer for three seconds. The study nurse counted 1-2-3 before starting, and also told the participant when to stop. Each test was performed three times with 30 seconds rest in-between, and the highest of the three measurements was used in the analyses.

### Laboratory measurements

Serum 25(OH)D was measured by an in-house developed liquid chromatography–tandem mass spectrometry method that detects both 25(OH)D_3_ and 25(OH)D_2_, as well as the sum of these presented as 25(OH)D in the results [22]. The laboratory takes part in the external quality program DEQAS. Plasma parathyroid hormone (PTH) was analyzed with an electrochemilumniscence immunoassay (ECLIA) using an automated clinical chemistry analyzer (Cobas 6000, Roche) with a reference range of 1.1–6.8 pmol/L for those <50 years and 1.1–7.5 pmol/L for those >50 years. Serum calcium was analyzed by an automated analyzer (Modular P, Roche Diagnostics, Mannheim, Germany).

### Statistical methods and power calculation

We used the statistical software package SPSS version 22. Descriptive results are shown as mean±SD if not stated otherwise. Normal distribution was evaluated by visual inspection of plots, and although there was some skewness and curtosis present, parametric tests were considered appropriate due to the large sample size. The end points were changes in muscle strength (delta strength) between the beginning and the end of the study (second and fourth visit), which was calculated separately for each participant. Independent t-tests were used for between-groups comparisons of delta strength for biceps, hip flexion, hand grip and pectoralis, as well as serum 25(OH)D, calcium and plasma PTH. Sensitivity analyses were performed in strata according to the randomization factors gender; vitamin D status in the Tromsø study (serum 25(OH)D above/below 25 nmol/L (10 ng/ml)); smoking status; and BMI above/below 27 kg/m^2^.

The power was calculated based on the main end point of the study, cardiovascular risk factors. The effect of vitamin D supplementation was assumed to be about two-third of the observed difference in cross-sectional studies between people with serum 25(OH)D levels of 30 and 80 nmol/l. Accordingly, between 300 and 500 participants were needed to attain a power of 0.8 and p<0.05 to detect a difference of 6.7 mmHg in systolic blood pressure, 3.3 mmHg in diastolic pressure, 0.26 mmol/l in triglycerides, 0.09 mmol/l in HDL-cholesterol and 0.57 in HOMA-IR. To account for an assumed drop-put rate of 16%, the aim was to include 600 subjects [19].

### Ethics

The study was approved by the Regional Committee for Medical Research Ethics (REK NORD 2013/1464) 4^th^ of September 2014 and by the Norwegian Medicines Agency (2013-003514-40). The study is registered at ClinicalTrials.gov NCT02750293. Due to a misunderstanding the study was registered there shortly after the first subject was included. The authors confirm that all ongoing and related trials for this drug/intervention are registered. The inclusion started 2^nd^ of June 2015, and last visit was performed 27^th^ of April 2017. All subjects gave their written informed consent.

## Results

**Fig 1:**
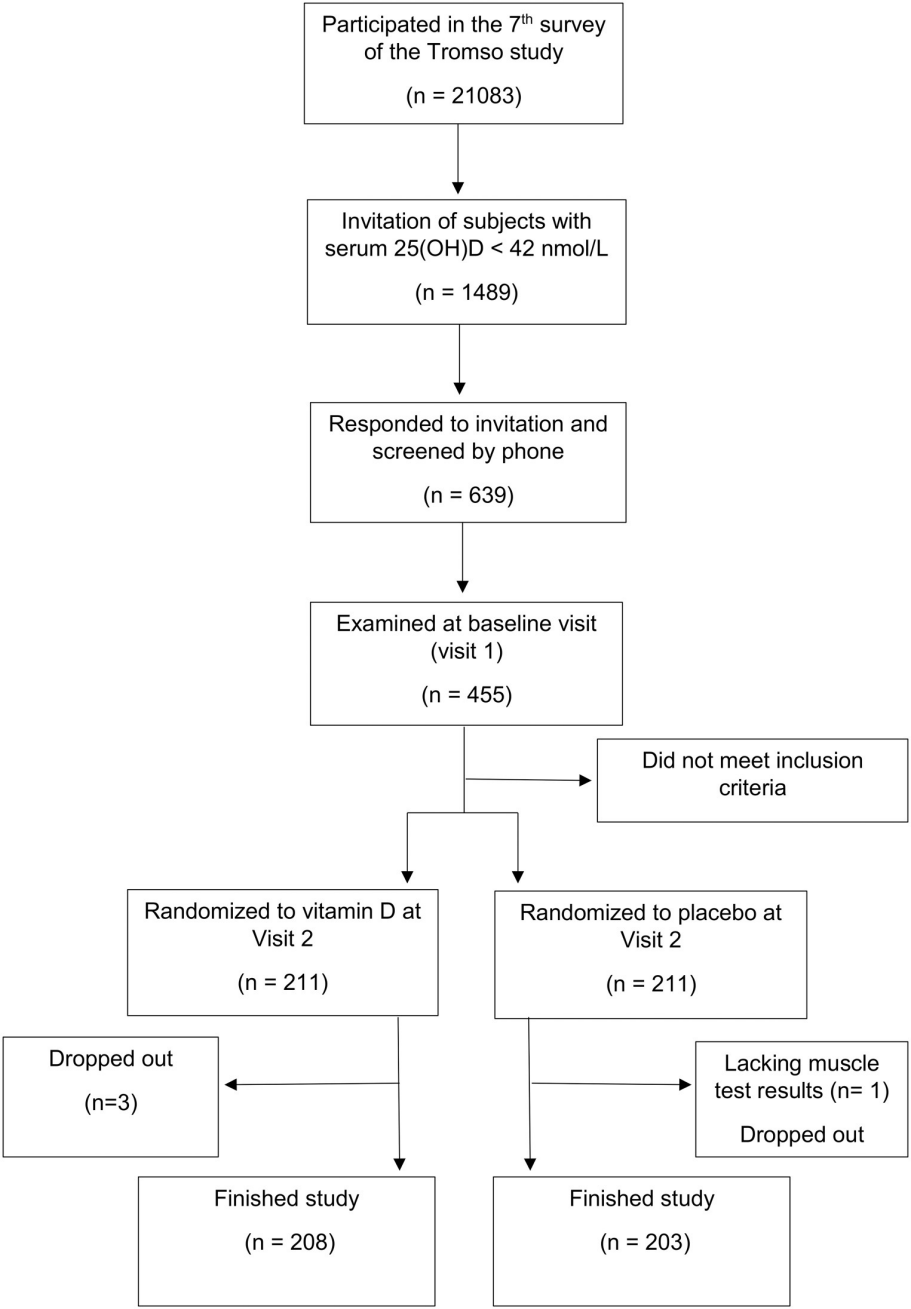
Flow chart of the inclusion of participants.

Of the 639 participants screened by phone, 455 attended the first visit with clinical examination and blood sampling. Thirty-three participants did not fulfill the inclusion criteria, and consequently 422 met to the second visit, and were randomized to vitamin D or placebo, one without successfully performed muscle testing. During the study, three participants withdrew their consent, and seven were excluded due to intercurrent disease or medication use not suitable with continuation. Thus, 411 completed the study and were included in the present analyses (Fig 1). Their baseline characteristics are presented in Table 1. Those randomized to vitamin D had a slightly lower 25(OH)D at baseline. Delta values for muscle strength, 25(OH)D, PTH and calcium levels are shown in Table 2 and compared between the treatment groups. Serum 25(OH)D increased significantly, accompanied by a significant decrease in PTH and a slight increase in serum calcium in the vitamin D group as compared to the placebo group. However, changes in muscle strength did not differ significant between the groups. The results for changes in muscle strength were the same in the sensitivity analyses in strata of gender; vitamin D; smoking status; and BMI as shown in Table 3.

**Table 1:** Baseline characteristics of the study population.

	Vitamin D __________________________	Placebo _________________________
n	208	203
Age (years)	51.5±8.6	52.6±8.7
Females (percentage)	46.6	46.8
BMI (kg/m^2^)	27.9±5.1	27.8±4.7
Serum 25(OH)D (nmol/L)*	32.6±11.1	35.1±13.6
Plasma PTH (pmol/L)	6.7±2.2	6.8±1.9
Serum calcium (mmol/L)	2.27±0.07	2.27±0.07
Smokers (percentage)	22.1	22.2
Hip flexion (N)	181.2±63.8	175.0±61.8
Biceps flexion (N)	129.5±66.8	129.4±66.8
Pectoralis (N)	307.7±137.3	306.0±138.5
Hand grip (kg)	41.8±11.6	41.9±12.7

N; Newton

Data are mean±SD if not stated otherwise.

*P for difference <0.05

**Table 2:** Changes in muscular strength during intervention.

	Vitamin D _______________________________		Placebo ______________________________		*P* for difference in change between groups
	n	Change	n	Change	
**Hip flexion (N)**	206	-0.6±49.9	198	-2.8±42.3	0.64
**Biceps flexion (N)**	206	5.2±49.3	198	1.2±51.1	0.43
**Pectoralis (N)**	205	18.4±83.4	197	11.9±82.6	0.43
**Hand grip (kg)**	206	1.0±4.2	199	0.5±4.3	0.31
**25(OH)D (nmol/L)**	208	56.2±22.2	203	-4.5±12.8	<0.01
**Plasma PTH (pmol/L)**	208	-0.8±1.4	203	0.5±1.5	<0.01
**Serum calcium (mmol/L)**	208	0.01±0.07	203	0.00±0.06	<0.01

N, Newton

Data are mean±SD.

**Table 3:** Changes in muscular strength during intervention. Stratified analyses.

	Vitamin D		Placebo		*P* for difference in change between groups	Vitamin D		Placebo		*P* for difference in change between groups
	n	change	n	change		n	change	n	change	
	**Female**					**Male**				
**Hip flexion (N)**	95	3.1±62.8	93	2.6±36.7	0.95	111	-2.8±62.5	105	-7.5±46.4	0.53
**Biceps flexion (N)**	95	5.2±29.7	93	12.9±36.0	0.11	111	5.1±61.5	105	-9.2±59.8	0.09
**Pectoralis (N)**	94	21.2±62.8	93	15.6±72.5	0.58	111	16.1±97.7	104	8.6±90.8	0.56
**Hand grip (kg)**	96	1.1±4.2	93	0.9±3.6	0.67	110	0.9±4.2	106	0.3±4.8	0.33
	**Serum 25(OH)D ≤25 nmol/l**					**Serum 25(OH)D>25 nmol/l**				
**Hip flexion (N)**	46	-1.0±57.9	44	-7.8±36.0	0.51	160	0.2±64.0	154	-1.3±43.9	0.80
**Biceps flexion (N)**	47	1.8±46.1	44	3.5±43.3	0.86	159	6.1±50.3	154	0.6±53.2	0.34
**Pectoralis (N)**	47	13.3±98.1	45	3.0±91.0	0.60	158	19.9±78.7	152	14.6±80.0	0.55
**Hand grip (kg)**	47	1.2±3.7	45	0.0±4.1	0.18	159	0.9±4.4	154	0.7±4.4	0.63
	**Non-smokers**					**Smokers**				
**Hip flexion (N)**	160	1.7±65.4	155	-5.1±39.3	0.26	46	-6.2±51.5	43	5.6±51.3	0.28
**Biceps flexion (N)**	161	6.8±49.2	155	-1.8±52.8	0.13	45	-0.7±49.8	43	12.2±43.5	0.20
**Pectoralis (N)**	160	22.2±74.9	155	19.8±83.4	0.79	45	5.1±108.3	42	-17.0±73.4	0.27
**Hand grip (kg)**	161	0.9±4.2	156	0.6±4.3	0.67	45	1.4±4.2	43	0.2±4.4	0.17
	**BMI<27 kg/m**^**3**^					**BMI≥27kg/m**^**3**^				
**Hip flexion (N)**	99	-3.1±78.1	96	0.7±46.9	0.68	105	2.6±44.0	102	-6.1±37.3	0.13
**Biceps flexion (N)**	99	6.1±52.1	93	1.4±52.5	0.53	105	4.5±47.3	105	1.1±50.1	0.61
**Pectoralis (N)**	99	12.0±92.7	92	9.1±78.5	0.82	104	24.3±74.2	105	14.4±86.3	0.38
**Hand grip (kg)**	100	1.0±3.7	94	0.5±4.1	0.39	105	0.8±4.6	105	0.6±4.5	0.67

N, Newton

Data are mean±SD.

The compliance to the study medication regimen was 100% in 86% of the participants, and above 84% in all. No serious study-related side effects were recorded. Two participants developed slight hypercalcemia; one male whose serum calcium normalized upon retesting, and one female who had developed primary hyperparathyroidism.

## Discussion

In this randomized controlled trial including adults with low serum 25(OH)D levels, no effects on muscular strength were observed after four months administration of vitamin D versus placebo. There were no gender differences.

These results are in contrast to a meta-analysis and review from 2014 concluding with a weak, but positive, effect on muscle strength with vitamin D supplementation [14]. The effect was only evident in persons aged 65 years or older, and the discrepancy with our results may therefore be explained by inclusion of younger persons in our study, with few participants >65 years of age (<10%). The expression of VDRs in muscle is reported to decrease with age [7], and therefore older people might be more vulnerable to vitamin D insufficiency. In a paper published after the meta-analysis, a risk reduction of nearly 50% for injurious falls needing medical attention was reported in a two year follow-up period after a two years intervention trial with vitamin D in older women [23]. However, in a recent nine months vitamin D intervention trial in older men with baseline 25(OH)D levels >60 nmol/l, no effects on gait speed or Short Physical Performance Battery were observed [24]. Another study found no effect on handgrip strength or Timed Up and Go in elderly people after administration of 12000, 24000 or 48000 IU/month cholecalciferol, for one year [25]. However, there was no placebo group. Finally, unfavorable effects on muscle strength were reported in elderly women randomized to receive 2800 IU/day cholecalciferol as compared to placebo for three months in a Danish study [26].

Several studies show that an effect of vitamin D supplementation on muscle strength is evident only in persons with serum 25(OH)D <30 nmol/l [14,27]. In contrast, we did not observe any effects in our subgroup of participants with baseline levels below a similar threshold (25 nmol/l), which is in accordance with another recently published meta-analysis which reported stronger effects of vitamin D supplementation on handgrip strength after exclusion of three studies with low baseline 25(OH)D levels. The authors speculated that the low levels represented patients with a higher degree of frailty, where it was too late to improve muscle strength [15]. In our study, the participants were community-dwelling, middle-aged people, recruited through a health survey, which are generally prone to healthy user bias. Also, only ten of the participants had sarcopenia as defined as maximal grip strength < 20 kg for women and 30 kg for men at baseline [28]. We therefore find it unlikely that the lack of effect could be explained by our participants being too frail to improve their muscle strength.

The effects of vitamin D may be dependent on interplay with factors like other conutrients [16], in particular calcium intake. Unfortunately, we did not have information regarding diet and calcium intake, but in general, the calcium intake in Scandinavian countries is high [29], and it might be that the threshold for clinical vitamin D deficiency is shifted downwards in such circumstances [30]. Also, some studies suggest that physical activity may interact with the effects of vitamin D on muscle function [31]. Unfortunately, we do not have available data of physical activity in our participants.

The heterogeneity in results between different studies and meta-analyses may at least to some part reflect differences in study design. It has been speculated on whether the dosing regimens might be of importance. Daily dosing has been argued to provide a more stable supply of cholecalciferol, and could act more physiologically than bolus dosing on a weekly or even more seldom basis, like used in the present study [32]. Another limitation of the present study could be that the duration of the intervention was only four months, and a longer duration may have been necessary to reveal any effects. However, for ethical reasons, it is challenging to perform long term studies including placebo groups in participants with documented vitamin D deficiency at baseline.

Also, we only measured one aspect of muscular function, namely strength, whereas endurance, power and balance were not assessed. We did, however, measure both the upper- and lower extremities, and included both proximal and distal strength. Still, it may be that we did not capture functional strength. For instance, one study among Brazilian postmenopausal women reported significant improvement in chair rising test, but no effect on handgrip strength [33].

Recently, it has been suggested that circulating levels of 1,25 dihydroxyvitamin D (1,25(OH)_2_D) might correlate better with muscle strength than 25(OH)D [34]. In a randomized six months trial both treatments resulted in muscle strength improvements, but administration of alphacalcidiol was significantly more effective than cholecalciferol [35]. It may therefore be that this metabolite should be taken into account. However, we did not have 1,25(OH)_2_D measurements available.

Finally, there were broad standard deviations in the changes in muscle strength in the treatment groups which limit the possibility of finding a significant difference in this medium-sized study. However, there were no trends in the results suggesting that this should explain the lack of effect of vitamin D.

The study also has several strengths. First, both 25(OH)D and outcomes were measured by validated methods. The participants were included based on low serum 25(OH)D measurements. The adherence to the study and the compliance rate were high, as confirmed by the substantially increase in 25(OH)D levels in the intervention group.

To summarize, our results do not support an effect of vitamin D on muscular strength. Future studies should consider using daily dosing, including assessment of conutrient status and physical activity, and exploring use of active vitamin D in order to achieve a greater understanding of the vitamin D metabolism and musculoskeletal health.

## Supporting information

S1 File CONSORT 2010 checklist. (DOC)

S2 File Final protocol. (DOCX)
