# Supplementary material for: Four months vitamin D supplementation to vitamin D insufficient individuals does not improve muscular strength: A randomized controlled trial
Source: PLoS One. 2019 Dec 16;14(12):e0225600. doi: 10.1371/journal.pone.0225600 (PMC6914329; doi:10.1371/journal.pone.0225600)
Supplement: S2 File — (DOCX) [file pone.0225600.s002.docx]

**The effect of vitamin D supplementation on cardiovascular risk factors in subjects with low serum 25-hydroxyvitamin D levels**

Version 6, 020516 (Protocol code number: TromsøEndo-2013-1)

EUDRACT NR: 2013-003514-40. Vitamin D tilskudd og risiko for hjerte-kar sykdom

Rolf Jorde, Elena Kamycheva, Johan Svartberg, Ragnar Joakimsen Guri Grimnes, Marie Kjærgaard, Yngve Figenschau, Ruth Paulssen, Jorid Degerstrøm, Arnfinn Andersen, Anne Sofie Furberg

*Tromsø Endocrine Research Group and Tromsø Gastroenterology Research Group, University of Tromsø, Norway.*

**Content**

| Summary, English and Norwegian | 2 |
| --- | --- |
| Introduction | 3 |
| Physiology of vitamin D | 3 |
| Vitamin D and disease states | 4 |
| Vitamin D, fractures and falls | 4 |
| Vitamin D and cardiovascular disease | 4 |
| Vitamin D and diabetes | 5 |
| Vitamin D and depression | 5 |
| Vitamin D and risk factors for disease | 5 |
| Vitamin D and bone mass density |  |
| Vitamin D and blood pressure | 5 |
| Vitamin D and lipids | 6 |
| Vitamin D and glucose metabolism and sRAGE | 6 |
| Vitamin D and inflammation | 6 |
| Vitamin D and arterial stiffness and pulse wave pressure | 7 |
| Vitamin D, muscle function, and cardio-respiratory fitness | 7 |
| Vitamin D and the gut mikrobiome | 7 |
| Ongoing large RCTs | 8 |
| Hypotheses; Rationale for choice of vitamin D dose; Project plan | 9 |
| Exclusion criteria | 10 |
| Analyses | 11 |
| Power calculations | 12 |
| Statistical analyses; Randomization procedure | 14 |
| Registration of adverse events; Management and Budget | 15 |
| Compliance with strategic documents, relevance to society | 15 |
| Ethical aspects and storage of data | 16 |
| Communication with users and exploitation of results; References | 17 |
| Attachments | 21 |
| Signatures | 23 |
| Amendment 1. Vitamin D and psoriasis | 24 |
| Amendment 2. Vitamin D and BMD | 30 |
| Amendment 3. Vitamin D and adipose tissue biopsies | 32 |
| Amendment 4. Vitamin D and sleep | 41 |

**Summary**

Vitamin D is a hormone with effects not only on the skeleton, but on most tissues in the body. Lack of vitamin D is associated with cardio-vascular disease (CVD) and type 2 diabetes, and also with risk factors for these diseases like hypertension, dyslipidemia, insulin resistance, and endothelial dysfunction. However, intervention studies with vitamin D have been inconclusive regarding diseases and risk factors. Most of these studies were done in white, Western populations in subjects fairly vitamin D sufficient, and accordingly, no benefits were to be expected. Also, in many studies the doses of vitamin D have been too low, and the studies underpowered. To firmly establish the role of vitamin D regarding CVD risk factors we will in the present study include 600 subjects with vitamin D deficiency (serum 25-hydroxyvitamin D (25(OH)D) < 30 nmol/L) and randomize to high dose vitamin D (3000 IU per day) versus placebo for four months. The subjects will be recruited based on 25(OH)D measurements in the forthcoming 7^th^ survey in the Tromsø study where more than 20 000 subjects are expected to attend. If our hypotheses are correct and the vitamin D supplement has a positive effect, this will be of great importance not only in countries with low sun exposure, but particularly for subjects in developing countries where vitamin D deficiency is highly prevalent.

**Norwegian summary**

Vitamin D er et hormon med effekt ikke bare på skjelettet, men på de fleste vev i kroppen. Mangel på vitamin D er assosiert med hjerte-kar sykdom og type 2 diabetes, samt med risikofaktorer for disse sykdommene så som hypertensjon, lipidforstyrrelser, insulin resistens og endotel dysfunksjon. Intervensjonsstudier med vitamin D har derimot så langt ikke vist sikker effekt på disse sykdommene og risikofaktorene. De fleste av disse studiene har vært gjort i hvite, vestlige populasjoner der deltagerne har hatt tilnærmet normal vitamin D status og en derfor ikke har kunnet forvente noen effekt. I tillegg har de doser av vitamin D som har vært benyttet trolig vært for lave og studiene for små. For med sikkerhet å etablere en rolle for vitamin D angående risikofaktorer for hjerte-kar sykdom, vil vi i denne studien inkludere 600 personer med vitamin D mangel (serum 25-hydroxyvitamin D (25(OH)D) < 30 nmol/L) og randomisere til høy dose vitamin D (3000 IU per dag) mot placebo i fire måneder. Personene vil bli rekruttert basert på 25(OH)D målinger i den kommende syvende Tromsøundersøkelsen hvor mer enn 20 000 personer forventes å delta. Hvis vår hypotese er korrekt og vitamin D tilskudd har positiv effekt, vil dette ha stor betydning ikke bare i land med lav sol eksponering, men spesielt i utviklingslandene der vitamin D mangel er svært utbredt.

**Introduction**

Citation from Editorial by Robert Heaney in the New England Journal of Medicine, 2012:

“There has been more ink spilled over the efficacy of vitamin D than over that of most nutrients, with the possible exception of sodium. Why is this? Dozens of randomized, controlled trials have been conducted — some large, and many small. Unfortunately, their results have been inconsistent — some positive, some null, and the odd one or two actually negative. Even the many available meta-analyses on the topic have yielded inconsistent results. If vitamin D is actually efficacious, why is there this inconsistency? ……It is clear that giving additional amounts of a nutrient to persons who already have enough, or not giving enough to push a person with a deficiency up onto the ascending limb of the response curve, is likely to produce a null response. …..We have continued to conduct trials (and include them in meta-analyses) without regard to ensuring the presence of two key features: baseline status and dose adequacy” (1).

There is no doubt that vitamin D is of vital importance for skeletal health with vitamin D deficiency in childhood leading to rickets and to osteomalacia and osteoporosis in adults (2). However, there are many indications for extra-skeletal effects of vitamin D which are to be expected as the vitamin D receptor (VDR) and the enzyme needed for local activation of vitamin D are found in numerous tissues (2). Furthermore, cross-sectional and longitudinal studies show a strong association between vitamin D deficiency and cardiovascular disease, cancer and diabetes; as well as risk factors for these diseases (2). However, whether there is a causal relation is still uncertain, which needs to be answered by properly performed randomized controlled trials (RCT). So far results from RCTs with vitamin D have generally been inconclusive for three reasons (1):

1. The subjects included have been vitamin D sufficient and accordingly no benefits were to be expected
2. The doses of vitamin D given have been too low
3. The studies have been underpowered

In the present study we will therefore include a large number of subjects with vitamin D deficiency and randomize to high dose vitamin D versus placebo to firmly establish an effect (if present) of vitamin D supplementation on cardiovascular risk factors. In view of the increasing focus on vitamin D in the media, increasing number of serum of 25-hydroxyvitamin D (25(OH)D) measurements performed in clinical practice, and increasing and uncritical use of vitamin D supplements, the present study is important and of high relevance not only for people living in the high North with limited sun exposure, but worldwide. In particular, it is relevant for older subjects where vitamin D deficiency is most frequently seen (2).

**Physiology of vitamin D**

Vitamin D is an ancient hormone with a multitude of functions. The main source of vitamin D is solar UV-radiation, and except for fatty fish there are few dietary sources (2). Both dietary and solar vitamin D undergo a hydroxylation in the liver to 25(OH)D which is the biochemical marker that best reflects the vitamin D status of the body (1). 25(OH)D serves as a substrate for 1-α-hydroxylase in the kidneys, which under tight regulation forms the active form of the vitamin, 1,25-dihydroxyvitamin D (1,25(OH)_2_ D). The main regulator is parathyroid hormone (PTH), which increases the formation of 1,25(OH)_2_ D as a response to low serum calcium levels. In addition, the enzyme 1-α-hydroxylase has also been found in extra-renal tissues, and activation of vitamin D can therefore occur locally (1). What regulates this local activation is not known.

In the circulation both 25(OH)D and 1,25(OH)_2_D are bound to plasma proteins, and less than 1% circulate in free form. The main binding protein is vitamin D binding protein (DBP) which accounts for more than 90 % of the vitamin D transportation. The active form of vitamin D binds to the VDR which is a nuclear receptor. This receptor has been found not only in the intestines, but in tissues throughout the body (1).

Vitamin D is essential for intestinal calcium absorption and plays a central role in maintaining calcium homeostasis and skeletal integrity. In accordance with the wide tissue distribution of 1-α-hydroxylase and VDR, vitamin D also has extra-skeletal effects. Thus, it appears to affect cell proliferation and inflammation, and low serum levels of 25(OH)D have been associated with a number of diseases (2). It has recently been shown that the serum levels of 25(OH)D and 1,25(OH)_2_D are not only determined by intake of vitamin D and sun exposure, but also by genetic factors (3). Furthermore, the function of the VDR is also genetically determined (4), which has to be taken into account when evaluating effects of vitamin D supplementation.

**Vitamin D and disease states.**

Vitamin D deficiency has been linked to osteoporosis, fractures, falls, cardiovascular disease (CVD), type 2 diabetes (T2DM), immunological diseases, cancer, cognitive decline, depression, infections and mortality (2).

Vitamin D, fractures and falls

The importance of vitamin D for bone is well known, and supplementation with vitamin D, at least when given together with calcium, increases bone density (5). A low serum 25(OH)D level is also a risk factor for future fractures (6), and a 30 % reduction in risk of hip fracture and 14 % reduction in risk for non-vertebral fracture are observed if giving 800 IU vitamin D per day or more (7). This might be explained through improved muscle function and prevention of falls as has been shown in RCTs (8).

Vitamin D and CVD

There is a clear association between vitamin D deficiency and risk of coronary heart disease. In a meta-analysis by Wang et al. including 19 prospective studies with 6123 CVD cases in 65 994 participants there was a 52 % increased risk of future CVD in those in the lowest compared to those in the highest serum 25(OH)D category (9). However, RCTs do not show a clear effect of vitamin D supplementation. Thus, in a meta-analysis by Elamin et al. including 51 RCTs they were unable to demonstrate a statistically significant reduction in mortality and CVD associated with vitamin D intake. However, the quality of the available evidence was low to moderate at best (10).

Vitamin D and diabetes

Vitamin D status may influence the risk of developing T2DM. In a meta-analysis by Khan et al. comprising 210 107 participants with 15 899 metabolic events, individuals in top versus bottom thirds of baseline 25(OH)D had a 20 % reduced risk of developing T2DM (11). However, large RCTs showing a convincing effect of vitamin D supplementation on glycemia and/or development of T2DM are still lacking, and the question “Vitamin D and diabetes, much ado about nothing?” was the title of a recent editorial on the subject (12).

Vitamin D and depression

The VDR is found in numerous cells in the CNS and lack of vitamin D and/or lack of VDR (VDR-knock out mice) have effects on behavior in animal studies (13). We have reported an association between low serum 25(OH)D and depression in the Tromsø study (14) which has been confirmed by others (15). We have also found a positive effect of vitamin D supplementation on depressive symptoms in one study (16); however, we were not able to reproduce that finding in a second one (17). Low levels of 25(OH)D are also associated with impaired cognitive function in cross-sectional and prospective studies, but RCT data are lacking (18).

Vitamin D and infections

A number of infections, like influenza, are more prevalent during the winter than the summer, and it has been suggested to associated with the vitamin D status (19). In-vitro studies support that vitamin D may have immune-stimulatory effects (20), and vitamin D may have a role as adjuvant therapy for tuberculosis (21).

We have previously found that there is as association between S. aureus nasal colonization and carriage and serum 25(OH)D levels (22). However, if this relation is causal is not known.

**Vitamin D and risk factors for disease.**

If there is a causal link between vitamin D deficiency and CVD and T2DM this could be mediated by an effect on known risk factors like blood pressure (BP), lipids, insulin resistance, endothelial function, cardio-respiratory fitness; and for fractures and falls, bone mass density (BMD) and muscle function.

Vitamin D and BMD

Reduced BMD is a major risk factor for future fractures. Although the importance of vitamin D for prevention of rickets is undisputed, the effect of vitamin D supplementation on BMD in subjects without profound vitamin D efficiency is questionable, as demonstrated in a recent review and meta-analysis (23). In our study the response in bone turnover markers, which can be considered a surrogate measure for effect on bone and is rapid, will be used to evaluate the effect on bone metabolism.

Vitamin D and BP

From animal studies it appears that vitamin D has an inhibitory effect on the renin-angiotensin system (24), and low serum 25(OH)D is associated with hypertension (25). RCTs have thus far failed to confirm an effect of vitamin D supplementation on BP (26). However, almost all studies on vitamin D and BP have been done using standard office BP, and it appears that central BP appears to be more sensitive to vitamin D supplementation than the peripheral BP (27).

Vitamin D and lipids

Similar as for BP, we have in the Tromsø study found low serum 25(OH)D to be associated with an unfavourable lipid profile (28). Furthermore, an increase in serum 25(OH)D was associated with a significant decrease in serum triglycerides (28). However, so far no convincing effects have been shown in intervention studies (29).

Vitamin D and glucose metabolism

With increasing serum 25(OH)D levels there is a decrease in HbA_1c_, fasting glucose and insulin (30). However, results from RCTs are non-conclusive. In the latest review and meat-analysis on this topic by George et al. that included 15 trials, no significant effect was seen on any measure of glucose metabolism in the studies combined (31).

Vitamin D and sRAGE

AGEs and their receptors are strongly implicated in the development of vascular diseases. When stimulated by AGEs, the receptors for AGEs (RAGEs) induce inflammation and are thought to fuel disease progression. These detrimental effects of RAGE may be counteracted by soluble RAGE (32). One RCT has reported increased serum sRAGE after supplementation with vitamin D which could therefore have a protective effect against the inflammatory action of AGE. This could possibly be another explanatory mechanism for vitamin D’s effects on chronic diseases (33).

Vitamin D and IFN-γ mediated inflammation

The pathogenesis of atherosclerosis is recognized as an inflammatory process and low levels of vitamin D have been linked to increased inflammation. Atherosclerotic plaques are infiltrated by activated macrophages and lymphocytes, contributing to disease progression. The monocyte-activating cytokine IFN-γ has been linked to acute atherosclerotic complications including major coronary events and death. As IFN-γ has a short half-life, other circulating markers (like kynurenine-to-tryptophan ratio (KTR) and neopterin), are often used as indicators of the activity of this cytokine (34). Vitamin D has been shown to supress IFN-γ mediated macrophage activation in vitro (35), and if replicated in vivo could be another explanatory mechanism for vitamin D’s actions.

There is a strong connection between the kynurenine metabolism and vitamin B6, as several enzymes in the kynurenine pathway require either B6 or B2 as co-factors. Pyridoxal 5`-phosphate (PLP) is the most commonly used serum marker of vitamin B6 status, and low levels of PLP are associated with inflammation. The vitamin B6 status therefore supplements the interpretation of changes in IFN-γ mediated macrophage activation (36).

Vitamin D and arterial stiffness and pulse wave velocity (PWV)

It is well established that vascular endothelial dysfunction and arterial stiffness precede and contribute to the development of CVD and predict long-term morbidity and mortality (37). Few studies have related this to vitamin D, but in a study by Al Mheid et al. where 554 subjects were included, impaired flow-mediated vaso-dilatiation and increased PWV was associated with vitamin D insufficiency (38). In the only related RCT so far, Sudgen et al. found that a single large dose of vitamin D improved flow mediated vasodilatation of the brachial artery in subjects with T2DM (39).

Vitamin D and muscle function

Vitamin D exerts a range of effects in skeletal muscle. Profound muscle weakness and changes in muscle morphology of adults with vitamin D deficiency have long been described. In clinical reports significant improvement in muscle strength in subjects with vitamin D myopathy is observed within three months after vitamin D substitution (40). Cross-sectional studies on muscle strength and serum 25(OH)D have in general showed a positive association (41, 42). Thus, for hand-grip the strength appears to increase with 0.09 kg per nmol/L, provided the serum 25(OH)D is below 60 nmol/L (43). However, RCTs are lacking (38).

Cardio-respiratory fitness

Poor cardio-respiratory fitness is a risk factor of CV morbidity and mortality (39). Maximal oxygen uptake is a recognized measure of cardiorespiratory fitness, and is inversely related to serum 25(OH)D levels (45, 46). However, so far there are no intervention studies with vitamin D published.

Vitamin D and the gut microbiome

The human gut microbiome (the gut flora) has been the focus of intense research the last decade due to the development of new techniques like genomic sequencing and array-based microbial identification. The microbiome has been shown to interact with the host in several ways in health and disease, including modulating the inflammatory host response to the gut, synthesizing small molecules and proteins that are taken up by the host, and changing the amount of available energy in the diet (47). A potential role for the microbiome in the development of several diseases, in particular auto-immune and allergic diseases and obesity, has been suggested (48).

Vitamin D is one potential regulator of the microbiome as well as the intestinal epithelial defense against infectious agents. Thus, Ooi et al. (49) demonstrated that mice without the VDR (knock-out mice) developed dysbiosis and greater susceptibility to injury in the gut, and Assa et al. (50) found vitamin D-deficient mice to have an altered composition of the fecal microbiome, increased colonic hyperplasia and epithelial barrier dysfunction. So far no studies on the effect of vitamin D in human gut microbiome has been published.

**Ongoing large RCTs**

Whether vitamin D deficiency is causally related to the above mentioned diseases is therefore uncertain. Similarly, there is no agreement on what are adequate or optimal serum 25(OH)D levels. This has been addressed by two recent reports; the first from the Institute of Medicine (IOM), at the request of the U.S. and Canadian governments, concluding that serum levels of 25(OH)D higher than 50 nmol/L have not been shown consistently to confer greater benefits (51); and the second as a response from the American Endocrine Society concluding that a 25(OH)D level above 75 nmol/L may have additional health benefits in reducing the risk of common cancers, autoimmune diseases, T2DM, CVD, and infectious diseases (52).

Both of these reviews, as practically every paper written about clinical effects of vitamin D, advocate that “more RCTs have to be performed”. An indeed, five large RCTs (The VITAL study in the US with 25 000 subjects, The FIND study in Finland with 18 000 subjects, The VIDA study from New Zealand with 5100 subjects, The European DOHealth study with 2150 subjects, and The VIDAL study from the UK with 20 000 subjects) with daily doses ranging from 2000 IU to 3200 IU are on the way to address key questions about vitamin D supplementation and health. The results are expected in 2017- 2020 (53).

However, it should be noted that in none of these large trials are vitamin D deficiency an inclusion criteria, and the study populations from where the participants are recruited are in general not at high risk for vitamin D deficiency. As pointed out by Pilz et al. in a letter published in Science: “Instead of performing these kinds of RCTs, the design of the ongoing vitamin D trials resembles previous (disappointing) vitamin trials, which attempted to establish a dose that should fit for the entire population. If the current vitamin D trials fail, we will ask ourselves why we did not perform RCTs exclusively in vitamin D–deficient patients rather than attempting to base conclusions on a heterogeneous population. Subgroup analyses of existing trials will not satisfy health authorities” (54).

Similarly, RCTs on surrogate endpoints like BP, lipids and insulin sensitivity have been inconclusive in spite of a considerable number of studies published, probably because the subjects included were not truly vitamin D deficient. According to the most recent meta-analyses, only one out of 10 studies on BP (26), three out of 10 studies on lipids (29), and one out of 15 studies on glucose metabolism (31) have included subjects with mean baseline serum 25(OH)D below 30 nmol/L. The reason for having included subjects with what many today would have called sufficient vitamin D levels, was the belief that the optimal serum 25(OH)D, based on ecological studies, should be between 100 and 150 nmol/L (55). In retrospect that was obviously wrong, and an effect (if present) will be most easily seen in those lacking the substance given.

**Hypothesis**

We believe that previous RCTs due to inclusion of vitamin sufficient subjects have masked important vitamin D effects. Our hypothesis is therefore:

***High dose vitamin D supplementation will improve CVD risk factors in subjects with vitamin D deficiency, improve muscle strength and cardio-respiratory fitness, mood, cognitive function, PWV and endothelial function, reduce the rate of S. aureus nasal colonization and carriage, and cause beneficial changes in the gut microbiome.***

The primary endpoints will be the difference in change between the vitamin D and placebo group in CVD risk factors (BP, lipid profile, insulin resistance) after four months.

The secondary endpoints will be:

- Difference in change in muscle strength (hand-grip, quadriceps, biceps, maximal oxygen uptake) after four months
- Difference in BDI score after four months
- Difference in cognitive function tests after four months
- Difference in PWV, augmentation index (AIX), and subendocardial viability ratio (SEVR) after four months
- Difference in S. aureus nasal colonization and carriage after four months
- Difference in gut microbiome after four months

The only way to show these effects is to perform the proper RCT.

**Rationale for choice of vitamin D dose**

Neither the “sufficient” nor the “optimal” serum 25(OH)D levels are known, but it is likely that the “therapeutic window” is wide. In order not to give a too low dose, and to stay well below what might be considered unhealthy (51), we will aim at a serum 25(OH)D level of 80 nmol/L. Based on our previous intervention studies (EUDRACT numbers 2004-004423-35, 2006-003186-14, 2007-001178-33, 2012-002082-35) where we have not seen any serious side effects with doses ranging from 3000 to 6000 IU vitamin D (cholecalciferol) per day, and as it does take some time for the serum 25(OH)D level to increase, we will start with a loading dose of 100.000 IU followed by 3000 IU per day. For the parameters in question we consider a four months intervention period sufficient.

**Project plan**

The Tromsø Study is a population based health survey that will be performed for the seventh time in 2015 (56). Approximately 30 000 subjects will be invited and at least 20 000 are expected to attend. We anticipate a consent rate of 50 % for the present study and will include 600 subjects (see power calculation). Accordingly, we will invite those with serum 25(OH)D < the 7^th^ percentile. In case the consent rate is lower than 50 %, we will increase the cut-off to the 10^th^ percentile.

Serum 25(OH)D will be measured consecutively (or weekly). The 7^th^ percentile for serum 25(OH)D cannot be set beforehand (the 25(OH)D assay in the last Tromsø survey in 2008 was an immunological method whereas in 2015 a LC-MSMS method will be used), and the 25(OH)D cut-off for invitation will be decided as the study moves along. Males and females 20 – 80 years old will be invited by mail with a short covering letter with the consent form enclosed (which they may keep as their own copy). If interested in participating, they will have to return the covering letter (where there will be a “yes” box to tick) in a prepaid envelope. If there is no response within two weeks a reminder will be sent. Those responding will then be contacted by a study nurse, given further information, asked about height and weight (to be used in the randomization stratification only), and if there are no contraindications in the medical history, an appointment for the first visit at the Clinical Research Unit at the University Hospital of North Norway will be made. At this first visit, in the morning after over-night fasting, the consent form will be signed, blood samples drawn, medical history (including medication) taken, questionnaires on vitamin D intake, physical activity, solar exposure, and Becks depression inventory (BDI) filled in, simple clinical tests performed, height, weight, hip and waist circumference and conventional BP measured. The subjects will be asked to bring a morning urine sample to this first visit. The subjects will be instructed on how to collect a stool sample that they are to bring with them to the next visit.

The second visit will be one of the following days with a second time conventional BP measurement, measurement of cognitive function, PWV, endothelial function and muscle strength, and nasal, throat and skin swab cultures for detection of S.aureus will be taken. If no contraindications from the medical history, clinical tests, BDI, and routine blood tests, the study drugs (capsules with 20 000 IU vitamin D or identically looking placebo) will be dispensed. While at the Clinical Research Unit the subject is to take five capsules and thereafter one capsule every week until the next visit after four months (average daily dose 3000 IU). We will use text message by mobile phone to remind the subjects to take the capsules. After eight weeks the subjects will be contacted by phone for registration of adverse events. After four months the same procedures as at baseline will be repeated and unused study drugs counted. At the end of the study all subjects (regardless of treatment allocation) will be advised to take vitamin D supplementation 800 IU per day and scheduled for measurement of serum 25(OH)D at the outpatient clinic after one year. During the study the subjects may not take vitamin D supplementation, including cod liver oil.

For measurement of maximal oxygen uptake we will include 200 subjects that are reasonably fit (preferably without BP medication and no cardiovascular disease). They will get an appointment for the treadmill exercise shortly after the second visit both at baseline and after four months. They will be given the loading dose after the treadmill exercise.

**Exclusion criteria**

- subjects allergic to nuts (the study capsules contain peanut oil)
- subjects with primary hyperparathyroidism
- granulomatous diseases (sarcoidosis, tuberculosis, Wegner’s granulomatosis)
- reduced kidney function (creatinine > 130 μmol/L in males and 120 μmol/L in females)
- systolic BP > 174 mmHg, diastolic BP > 104 mmHg,
- diabetes (fasting blood glucose > 6.9 mmol/L and/or HbA_1c_ > 6.5 %)
- renal stones the last five years
- subjects who use solarium on a regular basis (more than twice a month on average)
- subjects who plan holiday(s) in tropical areas (or Mediterranean during the summer months) for more than two weeks
- subjects with BDI score higher than 29
- subjects with clinical signs of proximal myopathy (problems with standing up from chair or walking stairs)
- subjects seriously ill and unfit for participation in a clinical study (as judged by one of the study doctors)
- subjects using vitamin D supplements exceeding 800 IU per day or active vitamin D drugs (Rocaltrol or Etalpha)

Females of child bearing potential (below the age of 50) may participate if they use acceptable methods of contraception (hormonal, IUD); if living in a relation with a partner who has been sterilized; if living in a lesbian relationship; or do not have or wish to have a male partner. If, in spite of the above, a pregnancy occurs during the study, that will lead to exclusion form the study. In females < 50 years a pregnancy test will be performed at inclusion.

.

If a subject during the study develops renal stones, serious disease that interferes with the intake of vitamin D, or a granulomatous disease, the subject will be excluded from the study. If clinical disease or pathological biochemical tests are found during the study, this will be followed up as clinically relevant.

**Analyses**

At inclusion and after four months fasting blood samples will be drawn for the following analyses:

- Hgb, SR, albumin, calcium, phosphate, creatinine, CK, HS-CRP, ASAT, ALAT, total cholesterol, HDL-cholesterol, LDL-cholesterol, triglycerides, Apolipoprotein A1, Apolipoprotein B, free thyroxine, TSH, glucose, HbA_1c_, insulin, C-peptide, s-RAGE, caboxy-methyllysin (CML, a robust glycosylation marker), 25(OH)D, DBP, PTH, bone formation and resorption markers (P1NP, CTX-1, BAP Leptin, Osteocalcin, OPN - Osteopontin, Osteoprotegerin, SOST, TNF-α, RANKL), serum markers of IFN-γ mediated macrophage activation and vitamin B6 status.
- As vitamin D functions through the VDR and gene transcription, an effect of vitamin D will be expressed in the amount mRNA in the tissues, and also in peripheral blood (57). Blood will therefore be drawn for measurement of RNA expression. At inclusion blood for DNA extraction will be drawn for determination of relevant vitamin D polymorphisms (3).

Additional serum and blood samples for back-up will be stored in the project-specific bio-bank for which the project leader Rolf Jorde will be responsible. Morning urine sample will be collected.

The vitamin D SNPs will be analyzed by KBioscience, UK, RNA microarray will be performed at The University of Tromsø, DBP will be analyzed by The Hormone Laboratory, Aker University Hospital, and all other analyses at the Department of Medical Biochemistry, University Hospital of North Norway.

Insulin sensitivity will be evaluated both with the HOMA-IR index and Quantitative Insulin Sensitivity Check Index (QUICKI) (58).

BP will be measured with an automatic device after a rest of 15 min. Three measurements will be made with 2 min in between. The mean of the last two will be used in the analyses. In addition, the central BP will be measured with the Pulsecor device (Uscom, Sydney, Australia) (27).

Depression will be evaluated with BDI (17), and cognitive function with The Twelve Word Memory Test, The Digit Symbol-Coding Test, and The Tapping Test (59).

Hand-grip, quadriceps and biceps muscle strength will be evaluated by hand held dynamometry (60).

Arterial stiffness and endothelial function will be evaluated with PWV, augmentation index (AIX), and subendocardial viability ratio (SEVR) using the Pulsecor device which also enables measurement of central blood pressure (27, 38).

Maximal oxygen uptake will be evaluated with graded treadmill exercise at the University of Tromsø in a sub-group of 200 subjects (61).

The nasal, throat and skin samples for S. aureus will be analyzed at the Department of Microbiology, University Hospital of North Norway.

The microbiome in the stool samples will be analyzed in batch at the end of the study in 2017. As the technology for these kind of analyses are rapidly changing, the method and laboratory will be decided later.

**Power calculations**

In Tromsø 7 some 30.000 subjects will be invited and an attendance rate of 75 % is a reasonable goal. Thus, at least 20 000 subjects will attend. We will include subjects with serum 25(OH)D < 7^th^ percentile which in the 6^th^ Tromsø study corresponded to 30 nmol/L. We will give a high dose of vitamin D to quickly raise the serum level to above 75 nmol/L (corresponding to the 75^th^ percentile), aiming at 80 nmol/L. If the difference in the various parameters between those with serum 25(OH)D < 30 nmol/L and those > 80 nmol/L is solely due to vitamin D, then this corresponds to the maximum effect one can achieve with vitamin D supplementation in vitamin D deficient subjects. However, a more realistic effect would be 2/3 of this difference. In the table below the SD for the endpoints, the differences in end point measure between low and high serum 25(OH)D, and the number needed if wanting a power of 0.8 and P < 0.05, are shown. Unfortunately, information on endpoint values corresponding to serum 25(OH)D < 30 and > 80 nmol/L is generally not available. Therefore, the best available information is used and the “low” and “high” levels which the calculations are based upon are shown in the table.

| **Parameter** | **Difference between low and high serum 25(OH)D** | **2/3**  **of**  **diff.** | **SD** | **N**  **needed**  **to**  **include** |
| --- | --- | --- | --- | --- |
| **Primary endpoints** |  |  |  |  |
| Office systolic BP (mmHg) (25)  Low 25(OH)D < 30, high 25(OH)D > 90 nmol/L | 10 | 6.7 | 24 | 450 |
| Office diastolic BP (mmHg) (25)  Low 25(OH)D < 30, high 25(OH)D > 90 nmol/L | 5 | 3.3 | 12 | 410 |
| Central systolic BP (mmHg) (27)  Low 25(OH)D < 56, high 25(OH)D > 107 nmol/L | 7 | 5 | 14 | 320 |
| Serum triglycerides (mmol/L) (28)  Low 25(OH)D < 25, high 25(OH)D > 74 nmol/L | 0.39 | 0.26 | 0.82 | 300 |
| Serum HDL-cholesterol (mmol/L) (28)  Low 25(OH)D < 25, high 25(OH)D > 74 nmol/L | 0.14 | 0.09 | 0.38 | 490 |
| Insulin resistance (HOMA) (62)  Low 25(OH)D < 10, high 25(OH)D > 90 percentil | 0.86 | 0.57 | 2.0 | 400 |
| **Secondary endpoints** |  |  |  |  |
| Muscle (knee extensor) (kg) (41)  Low 25(OH)D < 50, high 25(OH)D > 50 nmol/L | 1.8 | 1.2 | 2.6 | 150 |
| Muscle (hip flexor) (kg) (41)  Low 25(OH)D < 50, high 25(OH)D > 50 nmol/L | 2.6 | 1.7 | 3.0 | 100 |
| Muscle (hand grip) (kg) (63)  Low 25(OH)D < 25, high 25(OH)D > 50 nmol/L | 4.0 | 2.7 | 10 .0 | 420 |
| Maximal oxygen uptake (ml/kg/min) (45)  Low 25(OH)D < 50, high 25(OH)D > 75 nmol/L | 6.9 | 4.6 | 10.3 | 150 |
| Bone marker BALP (U/L) (64, 65)  Low 25(OH)D < 12, high 25(OH)D > 41 nmol/L | 4.5 | 3.0 | 10.4 | 400 |
| Arterial stiffness, PWV (m/sec) (38, 66)  Low 25(OH)D < 25, high 25(OH)D > 100 nmol/L | 0.75 | 0.50 | 2.0 | 500 |
| Arterial stiffness, PWV (m/sec) (67)  Low 25(OH)D < 50, high 25(OH)D > 50 nmol/L | 1.40 | 0.90 | 1.8 | 120 |

For arterial stiffness (PWV) two studies are included in the table since the published results differ considerably.

For depression we have previously found the BDI score to be significantly higher in subjects with serum 25(OH)D below than above 40 nmol/L in a study on 441 subjects and a slight but significant improvement after high dose vitamin D supplementation. As these scores are not normally distributed, a formal power calculation cannot be performed. For cognitive function we have no relevant data.

Based on the above we will need 500 subjects to complete the intervention to have a reasonable chance of demonstrating an effect of the vitamin D intervention on the primary endpoints. If assuming a drop-out rate of 16 %, we will have to include 600 subjects in the study. For testing of maximal oxygen uptake we will include 200 subjects.

These calculations are conservative as the SD are derived from more heterogeneous populations than the selected group we will include, and the difference in serum 25(OH)D levels between the low and high groups are for most parameters less than that we will have in the vitamin D group versus the placebo group after the intervention.

**Statistical analyses**

For between-group comparisons of placebo and vitamin D groups at baseline Student’s t-test, Mann-Whitney test or chi-square tests will be used. To compare the effect of vitamin D and placebo on the outcome variables, we will use ANCOVA models adjusting for the baseline value and relevant confounders (68), presenting the relative effect of vitamin D to the effect of placebo (set to 1 as reference). The results will be analysed both as intention-to-treat-analyses (with last observation carried forward) and per-protocol analyses. Tests for interactions between treatment group and sex, above or below serum 25(OH)D 25 nmol/L, and BMI will be performed for the primary outcome variable BP. Bonferroni corrections will be performed where appropriate, and *P* < 0.05 will be considered a significant finding.

**Randomization procedure**

The randomization will be stratified according to gender, vitamin D status (above/below 25 nmol/L), smoking status and BMI (< 27 kg/m^2^ and > 27 kg/m^2^, calculated from that reported on phone screening). The study nurse will give this information to the hospital’s randomization unit after the phone screening. Based on this the randomization unit will assign the subject with a randomization number using a block randomization procedure. This randomization number will be sent to the Clinical Research Unit (who do not have the randomization key) and to the Hospital Pharmacy (who do have the randomization key) and who will dispense the study medication accordingly. With this procedure the Hospital Pharmacy will have sufficient time to dispense and label the study medication before the subject comes to the second visit.

At the first visit the subject will sign the consent form. At the second visits the nurses at the Clinical Research Unit will pick up the study medication at the Hospital Pharmacy, by which time the results from the blood tests and BDI will be ready.

The subjects randomized to placebo will receive one box with placebo capsules; the subjects randomized to vitamin D will receive one box with vitamin D capsules. Each box will contain 25 capsules (each vitamin D capsule will contain 20 000 IU cholecalciferol (vitamin D3)). We will use vitamin D and placebo medication identical to that used in previously approved studies (EUDRACT numbers 2004-004423-35, 2006-003186-14, 2007-001178-33, 2012-002082-35). As a loading dose five capsules are to be taken before leaving the Clinical Research Unit at the second visit (or after the treadmill exercise). Thereafter one capsule is to be taken each week. The randomization number will follow the participant throughout the study.

**Registration of adverse events**

All adverse events will be registered on a specific form and kept in the subjects file at the Clinical Research Unit, University Hospital of North Norway (Attachment 1). A copy of the form will be sent to Elena Kamycheva for classification and if relevant, reporting to the health and ethics authorities (Attachment 2). At the telephone call after eight weeks the subjects will be asked for occurrence of adverse events.

**Management**

The study will be organized by the Tromsø Endocrine Research Group, headed by Professor Rolf Jorde (project leader). Tromsø Endocrine Research Group has had vitamin D as main focus the last eight years with 42 papers on vitamin D published in international journals. In particular, the group has performed five large RCTs with vitamin D and has one ongoing intervention study with 511 subjects with impaired glucose tolerance. The study will be performed at the Clinical Research Unit at the University Hospital of North Norway where there is more than 20 years of experience with RCTs.

Collaborators in the study:

- Elena Kamycheva MD PhD will have the study as her main post doc project and be the daily manager.
- Professor Johan Svartberg will be responsible for clinical evaluation of participants
- Ragnar Joakimsen MD, PhD will be responsible for the statistical analyses
- Guri Grimnes MD, PhD and Jorid Degerstrøm MD and Arnfinn Andersen will be responsible for the muscle testing
- Marie Kjærgaard MD, PhD will be responsible for the BDI and cognitive tests
- Professor Yngve Figenschau will be responsible for the biochemical analyses
- Professor Ruth Paulssen will be responsible for the RNA analyses
- Anne Sofie Furberg, PhD will be responsible for the S. Aureus and microbiome part of the study

**Budget**

The project will be financed by funds available to the Tromsø Endocrine Research Group

**Compliance with strategic documents and relevance to society**

At the University of Tromsø and the University Hospital of North Norway, studies on chronic diseases (and especially CVD) have top priority.

The prevalence of vitamin D deficiency is high worldwide (69). If we can demonstrate an effect on CVD risk factors in those deficient, this will be an important contribution to the ongoing discussion on vitamin D supplementation.

**Ethical aspects**

The project according to Protocol Version 3 has been approved by the Regional Ethics Committee and the National Medicines Agency. The study is approved to be performed at the Clinical Research Unit, University Hospital of North Norway.The present revised Version 4 will be submitted to the Regional Ethics Committee and the National Medicines Agency for approval. The study will follow the guidelines “Forskrift om klinisk utprøvning av legemidler til mennesker” FOR-2009-10-30-1321 and ICH guidelines E6 for GCP.

The study will be registered in ClinicalTrial.gov. A signed informed consent will be obtained from all subjects. The subjects will receive a gift card value 200 NKR to cover travel expenses in the study.

The present study does raise some important ethical questions as we will include and randomize subjects that according to Norwegian as well as international standards are vitamin D deficient (51, 52). However, these recommendations are based on cross-sectional studies, and at present there is no hard evidence that supplementation with vitamin D, except for the skeleton, will give health benefits. Also, vitamin D deficiency (even 25(OH)D levels < 15 nmol/L) is only associated with slightly elevated BP, modest dyslipidemia, modest insulin resistance and marginally increased scores on depression questionnaires like BDI. Accordingly, there is no rush to treat these subjects with vitamin D, unless they have overt clinical symptoms of vitamin D deficiency, which for practical purposes are restricted to proximal myopathia and severe depression.

Furthermore, we have with purpose chosen a short intervention period which for the parameters in question should be long enough to demonstrate an effect, and short enough to justify postponement of vitamin D supplementation in those randomized to placebo.

We will include the subjects as soon as possible after their visit in the Tromsø study by having the serum 25(OH)D measurements run as quickly as possible. In previous Tromsø studies the serum 25(OH)D measurements have been done in large batches and the results were therefore not available before a year after participation. Furthermore, without this RCT it is unlikely that serum 25(OH)D (which is an expensive analysis and financially linked to this RCT) will be measured at all.

And finally, all participants will be advised to take vitamin D after the intervention with a scheduled control after one year.

**Storage of data**

In the hospital records participation in the study and attendance at the visits will be registered as well as results of routine blood analyses. All data will be saved without name or personal identification number. A code list will connect the participants to the stored data. The code list and the data will be stored for 15 years after termination of the study and then destroyed.

**Communication with users and exploitation of results**

The publications will be written in cooperation between the members of the Tromsø Endocrine Research Group. Decision on authorship will be done by Rolf Jorde. Considering the size of the study and the general interest in vitamin D both in the scientific community and the general public, communications with users and exploitation of the results will not be a problem.

**References**

1. [Heaney RP](http://www.ncbi.nlm.nih.gov/pubmed?term=Heaney%20RP%5BAuthor%5D&cauthor=true&cauthor_uid=22762324). Vitamin D-baseline status and effective dose. [N Engl J Med](http://www.ncbi.nlm.nih.gov/pubmed/22762324) 2012; 367: 77-8

1. [Holick MF](http://www.ncbi.nlm.nih.gov/sites/entrez?Db=pubmed&Cmd=Search&Term=%22Holick%20MF%22%5BAuthor%5D&itool=EntrezSystem2.PEntrez.Pubmed.Pubmed_ResultsPanel.Pubmed_DiscoveryPanel.Pubmed_RVAbstractPlus). Vitamin D deficiency. [N Engl J Med](javascript:AL_get(this,%20'jour',%20'N%20Engl%20J%20Med.');) 2007; 357: 266-81
2. [Wang TJ](http://www.ncbi.nlm.nih.gov/pubmed?term=%22Wang%20TJ%22%5BAuthor%5D), et al. Common genetic determinants of vitamin D insufficiency: a genome-wide association study. [Lancet](javascript:AL_get(this,%20'jour',%20'Lancet.');) 2010; 376: 180-8
3. Raimondi S, et al. [Review and meta-analysis on vitamin D receptor polymorphisms and cancer risk.](http://www.ncbi.nlm.nih.gov/pubmed/19403841) Carcinogenesis 2009; 30: 1170-80
4. [Rizzoli R](http://www.ncbi.nlm.nih.gov/sites/entrez?Db=pubmed&Cmd=Search&Term=%22Rizzoli%20R%22%5BAuthor%5D&itool=EntrezSystem2.PEntrez.Pubmed.Pubmed_ResultsPanel.Pubmed_RVAbstractPlus) et al. The role of calcium and vitamin D in the management of osteoporosis. Bone 2008; 42: 246-9
5. [van Schoor NM](http://www.ncbi.nlm.nih.gov/sites/entrez?Db=pubmed&Cmd=Search&Term=%22van%20Schoor%20NM%22%5BAuthor%5D&itool=EntrezSystem2.PEntrez.Pubmed.Pubmed_ResultsPanel.Pubmed_DiscoveryPanel.Pubmed_RVAbstractPlus), et al. Vitamin D deficiency as a risk factor for osteoporotic fractures. [Bone](javascript:AL_get(this,%20'jour',%20'Bone.');) 2008; 42: 260-6
6. [Bischoff-Ferrari HA](http://www.ncbi.nlm.nih.gov/pubmed?term=Bischoff-Ferrari%20HA%5BAuthor%5D&cauthor=true&cauthor_uid=22762317), et al. A pooled analysis of vitamin D dose requirements for fracture prevention. [N Engl J Med](http://www.ncbi.nlm.nih.gov/pubmed/22762317) 2012; 367: 40-9
7. [Bischoff-Ferrari HA](http://www.ncbi.nlm.nih.gov/pubmed?term=Bischoff-Ferrari%20HA%5BAuthor%5D&cauthor=true&cauthor_uid=19797342), et al. Fall prevention with supplemental and active forms of vitamin D: a meta-analysis of randomised controlled trials. [BMJ](http://www.ncbi.nlm.nih.gov/pubmed/19797342) 2009; 339: b3692.
8. [Wang L](http://www.ncbi.nlm.nih.gov/pubmed?term=Wang%20L%5BAuthor%5D&cauthor=true&cauthor_uid=23149428), et al. Circulating 25-hydroxy-vitamin D and risk of cardiovascular disease: a meta-analysis of prospective studies. [Circ Cardiovasc Qual Outcomes](http://www.ncbi.nlm.nih.gov/pubmed/23149428) 2012; 5: 819-29
9. [Elamin MB](http://www.ncbi.nlm.nih.gov/pubmed?term=Elamin%20MB%5BAuthor%5D&cauthor=true&cauthor_uid=21677037), et al. Vitamin D and cardiovascular outcomes: a systematic review and meta-analysis. [J Clin Endocrinol Metab](http://www.ncbi.nlm.nih.gov/pubmed/21677037) 2011; 96: 1931-42
10. [Khan H](http://www.ncbi.nlm.nih.gov/pubmed?term=Khan%20H%5BAuthor%5D&cauthor=true&cauthor_uid=23107484), et al. Vitamin D, type 2 diabetes and other metabolic outcomes: a systematic review and meta-analysis of prospective studies. [Proc Nutr Soc](http://www.ncbi.nlm.nih.gov/pubmed/23107484) 2013; 72: 89-97
11. [Monnier L](http://www.ncbi.nlm.nih.gov/pubmed?term=%22Monnier%20L%22%5BAuthor%5D) & [Colette C](http://www.ncbi.nlm.nih.gov/pubmed?term=%22Colette%20C%22%5BAuthor%5D). Vitamin D and diabetes: Much ado about nothing? [Diabetes Metab](javascript:AL_get(this,%20'jour',%20'Diabetes%20Metab.');) 2010; 36: 323-5
12. [[Minasyan A](http://www.ncbi.nlm.nih.gov/pubmed?term=Minasyan%20A%5BAuthor%5D&cauthor=true&cauthor_uid=17482806), et al. Neophobia, sensory and cognitive functions, and hedonic responses in vitamin D receptor mutant mice. J Steroid Biochem Mol Biol](http://www.ncbi.nlm.nih.gov/pubmed/17482806) 2007; 104: 274-80.
13. [Kjærgaard M](http://www.ncbi.nlm.nih.gov/pubmed?term=Kj%C3%A6rgaard%20M%5BAuthor%5D&cauthor=true&cauthor_uid=21784535), et al. Low serum 25-hydroxyvitamin D levels are associated with depression in an adult Norwegian population. [Psychiatry Res](http://www.ncbi.nlm.nih.gov/pubmed/21784535) 2011; 190: 221-5.
14. [Anglin RE](http://www.ncbi.nlm.nih.gov/pubmed?term=Anglin%20RE%5BAuthor%5D&cauthor=true&cauthor_uid=23377209), et al. Vitamin D deficiency and depression in adults: systematic review and meta-analysis. [Br J Psychiatry](http://www.ncbi.nlm.nih.gov/pubmed/23377209) 2013; 202: 100-7
15. [Jorde R](http://www.ncbi.nlm.nih.gov/pubmed?term=Jorde%20R%5BAuthor%5D&cauthor=true&cauthor_uid=18793245), et al. Effects of vitamin D supplementation on symptoms of depression in overweight and obese subjects: randomized double blind trial. [J Intern Med](http://www.ncbi.nlm.nih.gov/pubmed/18793245) 2008; 264: 599-609
16. [Kjærgaard M](http://www.ncbi.nlm.nih.gov/pubmed?term=Kj%C3%A6rgaard%20M%5BAuthor%5D&cauthor=true&cauthor_uid=22790678), et al. Effect of vitamin D supplement on depression scores in people with low levels of serum 25-hydroxyvitamin D: nested case-control study and randomised clinical trial. [Br J Psychiatry](http://www.ncbi.nlm.nih.gov/pubmed/22790678) 2012; 201: 360-8
17. [van der Schaft J](http://www.ncbi.nlm.nih.gov/pubmed?term=van%20der%20Schaft%20J%5BAuthor%5D&cauthor=true&cauthor_uid=23727408), et al. The association between vitamin D and cognition: A systematic review. [Ageing Res Rev](http://www.ncbi.nlm.nih.gov/pubmed/23727408) 2013 pii: S1568-1637
18. [Cannell JJ](http://www.ncbi.nlm.nih.gov/sites/entrez?Db=pubmed&Cmd=Search&Term=%22Cannell%20JJ%22%5BAuthor%5D&itool=EntrezSystem2.PEntrez.Pubmed.Pubmed_ResultsPanel.Pubmed_DiscoveryPanel.Pubmed_RVAbstractPlus), et al. Epidemic influenza and vitamin D. [Epidemiol Infect.](javascript:AL_get(this,%20'jour',%20'Epidemiol%20Infect.');) 2006; 134:1129-40.
19. [Bikle DD](http://www.ncbi.nlm.nih.gov/sites/entrez?Db=pubmed&Cmd=Search&Term=%22Bikle%20DD%22%5BAuthor%5D&itool=EntrezSystem2.PEntrez.Pubmed.Pubmed_ResultsPanel.Pubmed_DiscoveryPanel.Pubmed_RVAbstractPlus). Vitamin D and the immune system: role in protection against bacterial infection.[Curr Opin Nephrol Hypertens.](javascript:AL_get(this,%20'jour',%20'Curr%20Opin%20Nephrol%20Hypertens.');) 2008; 17(4):348-52.
20. [Ralph AP](http://www.ncbi.nlm.nih.gov/sites/entrez?Db=pubmed&Cmd=Search&Term=%22Ralph%20AP%22%5BAuthor%5D&itool=EntrezSystem2.PEntrez.Pubmed.Pubmed_ResultsPanel.Pubmed_DiscoveryPanel.Pubmed_RVAbstractPlus), [Kelly PM](http://www.ncbi.nlm.nih.gov/sites/entrez?Db=pubmed&Cmd=Search&Term=%22Kelly%20PM%22%5BAuthor%5D&itool=EntrezSystem2.PEntrez.Pubmed.Pubmed_ResultsPanel.Pubmed_DiscoveryPanel.Pubmed_RVAbstractPlus), [Anstey NM](http://www.ncbi.nlm.nih.gov/sites/entrez?Db=pubmed&Cmd=Search&Term=%22Anstey%20NM%22%5BAuthor%5D&itool=EntrezSystem2.PEntrez.Pubmed.Pubmed_ResultsPanel.Pubmed_DiscoveryPanel.Pubmed_RVAbstractPlus) L-arginine and vitamin D: novel adjunctive immunotherapies in tuberculosis. [Trends Microbiol.](javascript:AL_get(this,%20'jour',%20'Trends%20Microbiol.');) 2008;16(7):336-44.
21. [Olsen K](http://www.ncbi.nlm.nih.gov/pubmed?term=Olsen%20K%5BAuthor%5D&cauthor=true&cauthor_uid=21811869), et al. Staphylococcus aureus nasal carriage is associated with serum 25-hydroxyvitamin D levels, gender and smoking status. The Tromsø Staph and Skin Study. [Eur J Clin Microbiol Infect Dis](http://www.ncbi.nlm.nih.gov/pubmed/21811869) 2012 ;31:465-73
22. [Reid IR](http://www.ncbi.nlm.nih.gov/pubmed?term=Reid%20IR%5BAuthor%5D&cauthor=true&cauthor_uid=24119980), et al. Effects of vitamin D supplements on bone mineral density: a systematic review and meta-analysis. [Lancet.](http://www.ncbi.nlm.nih.gov/pubmed/24119980) 2013 pii: S0140-6736(13)61647-5. doi: 10.1016/S0140-6736(13)61647-5. [Epub ahead of print]
23. [Li YC](http://www.ncbi.nlm.nih.gov/pubmed?term=Li%20YC%5BAuthor%5D&cauthor=true&cauthor_uid=15225806), et al. Vitamin D: a negative endocrine regulator of the renin-angiotensin system and blood pressure. [J Steroid Biochem Mol Biol](http://www.ncbi.nlm.nih.gov/pubmed?cmd=Search&doptcmdl=Citation&defaultField=Title%20Word&term=Li%5Bauthor%5D%20AND%20Vitamin%20D%3A%20a%20negative%20endocrine%20regulator%20of%20the%20renin%E2%80%93angiotensin%20system%20and%20blood%20pressure) 2004; 89-90: 387-92
24. [Jorde R](http://www.ncbi.nlm.nih.gov/pubmed?term=Jorde%20R%5BAuthor%5D&cauthor=true&cauthor_uid=20065152), et al. Serum 25-hydroxyvitamin D levels are strongly related to systolic blood pressure but do not predict future hypertension. [Hypertension](http://www.ncbi.nlm.nih.gov/pubmed/20065152) 2010; 55: 792-8
25. [Pittas AG](http://www.ncbi.nlm.nih.gov/pubmed?term=Pittas%20AG%5BAuthor%5D&cauthor=true&cauthor_uid=20194237), et al. Systematic review: Vitamin D and cardiometabolic outcomes. [Ann Intern Med](http://www.ncbi.nlm.nih.gov/pubmed/20194237) 2010; 152: 307-14
26. [Larsen T](http://www.ncbi.nlm.nih.gov/pubmed?term=Larsen%20T%5BAuthor%5D&cauthor=true&cauthor_uid=22854639), et al. Effect of cholecalciferol supplementation during winter months in patients with hypertension: a randomized, placebo-controlled trial. [Am J Hypertens.](http://www.ncbi.nlm.nih.gov/pubmed/22854639##) 2012, 25:1215-22.
27. [Jorde R](http://www.ncbi.nlm.nih.gov/pubmed?term=Jorde%20R%5BAuthor%5D&cauthor=true&cauthor_uid=20823896), et al. High serum 25-hydroxyvitamin D concentrations are associated with a favorable serum lipid profile. [Eur J Clin Nutr](http://www.ncbi.nlm.nih.gov/pubmed/20823896) 2010; 64: 1457-64.
28. [Jorde R](http://www.ncbi.nlm.nih.gov/pubmed?term=Jorde%20R%5BAuthor%5D&cauthor=true&cauthor_uid=21640757) & [Grimnes G](http://www.ncbi.nlm.nih.gov/pubmed?term=Grimnes%20G%5BAuthor%5D&cauthor=true&cauthor_uid=21640757). Vitamin D and metabolic health with special reference to the effect of vitamin D on serum lipids. [Prog Lipid Res](http://www.ncbi.nlm.nih.gov/pubmed/21640757) 2011; 50: 303-12
29. [Hutchinson MS](http://www.ncbi.nlm.nih.gov/pubmed?term=Hutchinson%20MS%5BAuthor%5D&cauthor=true&cauthor_uid=21517716), et al. Serum 25-hydroxyvitamin D levels are inversely associated with glycated haemoglobin (HbA(1c)). The Tromsø Study. [Scand J Clin Lab Invest](http://www.ncbi.nlm.nih.gov/pubmed/21517716) 2011; 71: 399-406
30. [George PS](http://www.ncbi.nlm.nih.gov/pubmed?term=George%20PS%5BAuthor%5D&cauthor=true&cauthor_uid=22486204), et al. Effect of vitamin D supplementation on glycaemic control and insulin resistance: a systematic review and meta-analysis. [Diabet Med](http://www.ncbi.nlm.nih.gov/pubmed/22486204) 2012; 29: e142-50
31. [Selvin E](http://www.ncbi.nlm.nih.gov/pubmed/?term=Selvin%20E%5BAuthor%5D&cauthor=true&cauthor_uid=23396398) et al. sRAGE and risk of diabetes, cardiovascular disease, and death. Diabetes 2013 62:2116-21
32. [Irani M](http://www.ncbi.nlm.nih.gov/pubmed/?term=Irani%20M%5BAuthor%5D&cauthor=true&cauthor_uid=24606102) et al. Vitamin D increases serum levels of the soluble receptor for advanced glycation end products in women with PCOS. J Clin Endocrinol Metab 2014 99:E886-90
33. Pedersen ER, et al. Systemic markers of interferon-gamma-mediated immune activation and long-term prognosis in patients with stable coronary artery disease. Arterioscler Thromb Vasc Biol 2011; 31: 698-704
34. Helming L, et al. 1alpha,25-Dihydroxyvitamin D3 is a potent suppressor of interferon gamma-mediated macrophage activation. Blood 2005; 106: 4351-8
35. Christensen MH, et al. Vitamin B6 status and interferon-gamma-mediated immune activation in primary hyperparathyroidism. J Intern Med 2012; 272: 583-91
36. [Weber T](http://www.ncbi.nlm.nih.gov/pubmed?term=Weber%20T%5BAuthor%5D&cauthor=true&cauthor_uid=18787521), et al. Arterial stiffness and arterial wave reflections are associated with systolic and diastolic function in patients with normal ejection fraction. [Am J Hypertens](http://www.ncbi.nlm.nih.gov/pubmed/18787521) 2008; 21: 1194-202
37. [Al Mheid I](http://www.ncbi.nlm.nih.gov/pubmed?term=Al%20Mheid%20I%5BAuthor%5D&cauthor=true&cauthor_uid=21718915), et al. Vitamin D status is associated with arterial stiffness and vascular dysfunction in healthy humans. [J Am Coll Cardiol](http://www.ncbi.nlm.nih.gov/pubmed/21718915) 2011; 58: 186-92
38. [Sugden JA](http://www.ncbi.nlm.nih.gov/pubmed?term=Sugden%20JA%5BAuthor%5D&cauthor=true&cauthor_uid=18279409), et al. Vitamin D improves endothelial function in patients with Type 2 diabetes mellitus and low vitamin D levels. [Diabet Med](http://www.ncbi.nlm.nih.gov/pubmed/18279409) 2008; 25: 320-5
39. [Girgis CM](http://www.ncbi.nlm.nih.gov/pubmed?term=Girgis%20CM%5BAuthor%5D&cauthor=true&cauthor_uid=23169676), et al. The roles of vitamin D in skeletal muscle: form, function, and metabolism. [Endocr Rev](http://www.ncbi.nlm.nih.gov/pubmed/23169676) 2013; 34: 33-83
40. [Mastaglia SR](http://www.ncbi.nlm.nih.gov/pubmed?term=Mastaglia%20SR%5BAuthor%5D&cauthor=true&cauthor_uid=21528160), et al. Effect of vitamin D nutritional status on muscle function and strength in healthy women aged over sixty-five years. [J Nutr Health Aging](http://www.ncbi.nlm.nih.gov/pubmed/21528160?dopt=Abstract##) 2011; 15: 349-54
41. [Annweiler C](http://www.ncbi.nlm.nih.gov/pubmed?term=Annweiler%20C%5BAuthor%5D&cauthor=true&cauthor_uid=19214335), et al. Is there an association between serum 25-hydroxyvitamin D concentration and muscle strength among older women? Results from baseline assessment of the EPIDOS study. [J Nutr Health Aging](http://www.ncbi.nlm.nih.gov/pubmed/19214335) 2009; 13: 90-5
42. [Janssen HC](http://www.ncbi.nlm.nih.gov/pubmed?term=Janssen%20HC%5BAuthor%5D&cauthor=true&cauthor_uid=23810236), et al. Vitamin D and muscle function: is there a threshold in the relation? [J Am Med Dir Assoc.](http://www.ncbi.nlm.nih.gov/pubmed/23810236##) 2013, 14:627.e13-8.
43. [Lyerly GW](http://www.ncbi.nlm.nih.gov/pubmed?term=Lyerly%20GW%5BAuthor%5D&cauthor=true&cauthor_uid=19720775), et al. The association between cardiorespiratory fitness and risk of all-cause mortality among women with impaired fasting glucose or undiagnosed diabetes mellitus. [Mayo Clin Proc.](http://www.ncbi.nlm.nih.gov/pubmed/19720775) 2009 84:780-6.
44. [Ardestani A](http://www.ncbi.nlm.nih.gov/pubmed?term=Ardestani%20A%5BAuthor%5D&cauthor=true&cauthor_uid=21349488), et al. Relation of vitamin D level to maximal oxygen uptake in adults. [Am J Cardiol.](http://www.ncbi.nlm.nih.gov/pubmed/21349488) 2011107:1246-9.
45. [Mowry DA](http://www.ncbi.nlm.nih.gov/pubmed?term=Mowry%20DA%5BAuthor%5D&cauthor=true&cauthor_uid=19861594), et al. Association among cardiorespiratory fitness, body fat, and bone marker measurements in healthy young females. [J Am Osteopath Assoc.](http://www.ncbi.nlm.nih.gov/pubmed/?term=mowry+da+and+heelan+ka) 2009, 109:534-9.
46. [Sweeney TE](http://www.ncbi.nlm.nih.gov/pubmed?term=Sweeney%20TE%5BAuthor%5D&cauthor=true&cauthor_uid=23571517), [Morton JM](http://www.ncbi.nlm.nih.gov/pubmed?term=Morton%20JM%5BAuthor%5D&cauthor=true&cauthor_uid=23571517). The human gut microbiome: a review of the effect of obesity and surgically induced weight loss. [JAMA Surg](http://www.ncbi.nlm.nih.gov/pubmed/23571517) 2013;148:563-9
47. [Cénit MC](http://www.ncbi.nlm.nih.gov/pubmed?term=C%C3%A9nit%20MC%5BAuthor%5D&cauthor=true&cauthor_uid=24882755), [Matzaraki V](http://www.ncbi.nlm.nih.gov/pubmed?term=Matzaraki%20V%5BAuthor%5D&cauthor=true&cauthor_uid=24882755), [Tigchelaar EF](http://www.ncbi.nlm.nih.gov/pubmed?term=Tigchelaar%20EF%5BAuthor%5D&cauthor=true&cauthor_uid=24882755), [Zhernakova A](http://www.ncbi.nlm.nih.gov/pubmed?term=Zhernakova%20A%5BAuthor%5D&cauthor=true&cauthor_uid=24882755). Rapidly expanding knowledge on the role of the gut microbiome in health and disease. [Biochim Biophys Acta](http://www.ncbi.nlm.nih.gov/pubmed/24882755) 2014. pii: S0925-4439(14)00151-3.
48. [Ooi JH](http://www.ncbi.nlm.nih.gov/pubmed?term=Ooi%20JH%5BAuthor%5D&cauthor=true&cauthor_uid=23966330), [Li Y](http://www.ncbi.nlm.nih.gov/pubmed?term=Li%20Y%5BAuthor%5D&cauthor=true&cauthor_uid=23966330), [Rogers CJ](http://www.ncbi.nlm.nih.gov/pubmed?term=Rogers%20CJ%5BAuthor%5D&cauthor=true&cauthor_uid=23966330), [Cantorna MT](http://www.ncbi.nlm.nih.gov/pubmed?term=Cantorna%20MT%5BAuthor%5D&cauthor=true&cauthor_uid=23966330). Vitamin D regulates the gut microbiome and protects mice from dextran sodium sulfate-induced colitis. [J Nutr](http://www.ncbi.nlm.nih.gov/pubmed/23966330) 2013;143:1679-86
49. [Assa A](http://www.ncbi.nlm.nih.gov/pubmed?term=Assa%20A%5BAuthor%5D&cauthor=true&cauthor_uid=24755435), et al. Vitamin D Deficiency Promotes Epithelial Barrier Dysfunction and Intestinal Inflammation. [J Infect Dis.](http://www.ncbi.nlm.nih.gov/pubmed/24755435) 2014 Apr 21. pii: jiu235. [Epub ahead of print]
50. [Ross AC](http://www.ncbi.nlm.nih.gov/pubmed?term=Ross%20AC%5BAuthor%5D&cauthor=true&cauthor_uid=21118827), et al. The 2011 report on dietary reference intakes for calcium and vitamin D from the Institute of Medicine: what clinicians need to know. [J Clin Endocrinol Metab.](http://www.ncbi.nlm.nih.gov/pubmed/21118827) 2011;96:53-8.
51. [Holick MF](http://www.ncbi.nlm.nih.gov/pubmed?term=Holick%20MF%5BAuthor%5D&cauthor=true&cauthor_uid=21646368), et al. Evaluation, treatment, and prevention of vitamin D deficiency: an Endocrine Society clinical practice guideline. [J Clin Endocrinol Metab](http://www.ncbi.nlm.nih.gov/pubmed/21646368) 2011; 96: 1911-30
52. [Kupferschmidt](http://www.sciencemag.org/search?author1=Kai+Kupferschmidt&sortspec=date&submit=Submit) K. Uncertain Verdict as Vitamin D Goes On Trial.Science 2012: 337: 1476-1478
53. [Pilz S](http://www.ncbi.nlm.nih.gov/pubmed?term=Pilz%20S%5BAuthor%5D&cauthor=true&cauthor_uid=23161977), et al. Disease prevention: vitamin D trials. [Science](http://www.ncbi.nlm.nih.gov/pubmed/23161977) 2012; 338: 883
54. [Vieth R](http://www.ncbi.nlm.nih.gov/pubmed?term=Vieth%20R%5BAuthor%5D&cauthor=true&cauthor_uid=16766239). What is the optimal vitamin D status for health? [Prog Biophys Mol Biol.](http://www.ncbi.nlm.nih.gov/pubmed/16766239) 2006;92:26-32.
55. [Jacobsen BK](http://www.ncbi.nlm.nih.gov/pubmed?term=Jacobsen%20BK%5BAuthor%5D&cauthor=true&cauthor_uid=21422063), et al. Cohort profile: the Tromso Study. [Int J Epidemiol](http://www.ncbi.nlm.nih.gov/pubmed/21422063) 2012; 41: 961-7
56. [de Mello VD](http://www.ncbi.nlm.nih.gov/pubmed?term=de%20Mello%20VD%5BAuthor%5D&cauthor=true&cauthor_uid=18191048), et al. Effect of weight loss on cytokine messenger RNA expression in peripheral blood mononuclear cells of obese subjects with the metabolic syndrome. [Metabolism](http://www.ncbi.nlm.nih.gov/pubmed/18191048) 2008; 57: 192-9
57. Katz A, et al. Quantitative Assessment Check Index: A simple accurate method for assessing insulin sensitivity in humans. J Clin Endocrinol Metab 2000; 85: 2402-10
58. [Arntzen KA](http://www.ncbi.nlm.nih.gov/pubmed?term=Arntzen%20KA%5BAuthor%5D&cauthor=true&cauthor_uid=22222422), et al. Carotid atherosclerosis predicts lower cognitive test results: a 7-year follow-up study of 4,371 stroke-free subjects - the Tromsø study. [Cerebrovasc Dis](http://www.ncbi.nlm.nih.gov/pubmed/22222422) 2012; 33: 159-65
59. [Stark T](http://www.ncbi.nlm.nih.gov/pubmed?term=Stark%20T%5BAuthor%5D&cauthor=true&cauthor_uid=21570036), et al. Hand-held dynamometry correlation with the gold standard isokinetic dynamometry: a systematic review. [PM R](http://www.ncbi.nlm.nih.gov/pubmed/21570036) 2011; 3: 472-9
60. [Emaus A](http://www.ncbi.nlm.nih.gov/pubmed?term=Emaus%20A%5BAuthor%5D&cauthor=true&cauthor_uid=21062845), et al. Does a variation in self-reported physical activity reflect variation in objectively measured physical activity, resting heart rate, and physical fitness? Results from the Tromso study. [Scand J Public Health](http://www.ncbi.nlm.nih.gov/pubmed/21062845) 2010;38(5 Suppl):105-18
61. [Hutchinson MS](http://www.ncbi.nlm.nih.gov/pubmed?term=Hutchinson%20MS%5BAuthor%5D&cauthor=true&cauthor_uid=22419202), et al. Serum 25-hydroxyvitamin D levels in subjects with reduced glucose tolerance and type 2 diabetes - the Tromsø OGTT-study. Int J Vitam Nutr Res. 2011 Sep;81(5):317-27.
62. [Houston DK](http://www.ncbi.nlm.nih.gov/pubmed?term=Houston%20DK%5BAuthor%5D&cauthor=true&cauthor_uid=17452740), et al. Association between vitamin D status and physical performance: the InCHIANTI study. [J Gerontol A Biol Sci Med Sci.](http://www.ncbi.nlm.nih.gov/pubmed/?term=houston+dk+and+cecari+m) 2007, 62:440-6.
63. [Lowe NM](http://www.ncbi.nlm.nih.gov/pubmed?term=Lowe%20NM%5BAuthor%5D&cauthor=true&cauthor_uid=20102676), et al. Vitamin D status and markers of bone turnover in Caucasian and South Asian postmenopausal women living in the UK. [Br J Nutr.](http://www.ncbi.nlm.nih.gov/pubmed/20102676) 2010 103:1706-10.
64. [Ikeda T](http://www.ncbi.nlm.nih.gov/pubmed?term=Ikeda%20T%5BAuthor%5D&cauthor=true&cauthor_uid=24313921), et al. Effects of alendronate or alfacalcidol on bone metabolic indices and bone mineral density in patients with ophthalmologic disease treated with glucocorticoid. [Mod Rheumatol.](http://www.ncbi.nlm.nih.gov/pubmed/24313921) 2013 Dec 9. [Epub ahead of print]
65. [Giallauria F](http://www.ncbi.nlm.nih.gov/pubmed?term=Giallauria%20F%5BAuthor%5D&cauthor=true&cauthor_uid=22767638), et al. Arterial stiffness and vitamin D levels: the Baltimore longitudinal study of aging. [J Clin Endocrinol Metab](http://www.ncbi.nlm.nih.gov/pubmed/22767638) 2012; 97: 3717-23
66. [Seker T](http://www.ncbi.nlm.nih.gov/pubmed?term=Seker%20T%5BAuthor%5D&cauthor=true&cauthor_uid=23867332), et al. Serum 25-hydroxyvitamin D is associated with both arterial and ventricular stiffness in healthy subjects. [J Cardiol.](http://www.ncbi.nlm.nih.gov/pubmed/23867332) 2013, 62:361-5.
67. Vickers AJ, Altman DG. Statistics notes: Analysing controlled trials with baseline and follow-up measurements. BMJ 2001;10:1123-1124
68. [van Schoor NM](http://www.ncbi.nlm.nih.gov/pubmed?term=van%20Schoor%20NM%5BAuthor%5D&cauthor=true&cauthor_uid=21872807) & [Lips P](http://www.ncbi.nlm.nih.gov/pubmed?term=Lips%20P%5BAuthor%5D&cauthor=true&cauthor_uid=21872807). Worldwide vitamin D status. [Best Pract Res Clin Endocrinol Metab](http://www.ncbi.nlm.nih.gov/pubmed/21872807) 2011; 25: 671-80

Attachment 1

**Registrering av uønskede medisinske hendelser i studien** **”The effect of vitamin D supplementation on cardiovascular risk factors in subjects with low serum 25-hydroxyvitamin D levels”**

Dersom det opptrer uønskede medisinske hendelser skal dette alltid registreres og dette skjema fylles ut, uansett om hendelsen kan relateres til studiemedisinen eller ikke, og uansett alvorsgrad av hendelsen. Det fylles ut ett skjema for hver hendelse.

**Kopi av utfylt skjema sendes til Elena Kamycheva** og original oppbevares i pasientmappen på Forskningsposten. Elena Kamycheva fyller ut eget skjema der hendelsen klassifiseres og tiltak anføres. Arkiveres i egen perm på Elena Kamycheva sitt kontor.

Det printes ut laboratoriesvar dersom noen av blod/urin prøvene tatt i forbindelse med (og etter oppstart av) studien faller utenfor laboratoriets normalområde.

- Dersom avvikende svar ikke er klinisk signifikant anføres dette på utskrift og sendes forskningspost for arkivering i CRF.
- Dersom avvikende svar er klinisk signifikant, og årsaken/hendelsen til avvikende svar ikke allerede er registrert i vanlig event rapport, lages det egen event rapport som sammen med lab utskrift sendes for arkivering i CRF. I tillegg fylles ut klassifikasjon av hendelsen som sammen med kopi av event rapport arkiveres hos Elena Kamycheva.
- Ansvar for registrering av avvikende blodprøvesvar påfaller Elena Kamycheva.

Dersom det registreres en alvorlig medisinsk hendelse skal dette meddeles Rolf Jorde (som i denne sammenheng fungerer som sponsor) og Elena Kamycheva umiddelbart. Alvorlig medisinsk hendelse i denne sammenheng defineres som: - Død - livstruende sykdom - hospitalisering - vedvarende uførhet eller sykdom - serum kalsium > 2.80 mmol/L

Navn ………….………………….……………… f. dato…………… Personkode……………

**Beskrivelse av hendelsen (bruk ev. eget ark)** Dato for når plagene/hendelsen startet:……….

Utfylt av:……………………………………………… Dato:……………………….

Attachment 2

**Klassifikasjon og tiltak ved uønskete medisinsk hendelser i studien** **“The effect of vitamin D supplementation on cardiovascular risk factors in subjects with low serum 25-hydroxyvitamin D levels”**

Navn …………….. ………………….……… ……f. dato…………..Person kode …………

Kort resymé av hendelsen:

Medisinske tiltak:

Mulig relasjon til studiemedisin: Ja Nei Medikamentkode brutt Ja Nei Konferert med Rolf Jorde (sponsor) Ja Nei Fortsetter i studien Ja Nei

Hendelsen klassifisert som: Alvorlig uønsket hendelse Alvorlig uønsket virkning Uønsket hendelse Uønsket virkning

Alvorlige uønskede virkninger meldes sponsor umiddelbart og SLV, REK V, EMEAs EudraVigilance database og FP innen 15 kalender dager. Alvorlig medisinsk hendelse i denne sammenheng defineres som: - Død - livstruende sykdom - hospitalisering - vedvarende uførhet eller sykdom - serum kalsium > 2.80 mmol/L.

Dødsfall eller livstruende hendelser meldes sponsor umiddelbart og de øvrige innen 7 dager hvis klassifisert som ”virkning” (sammenheng eller mulig sammenheng med utprøvningsmedikament).

Meldt til SLV Ja Nei Meldt til Styret Forskningsposten (FP) Ja Nei Meldt til REK V Ja Nei Meldt til UNN (pasientskadeerstatning) Ja Nei Meldt til EMEAs EudraVigilance database Ja Nei

Underskrift av lege som har vurdert hendelsen……………………… Dato……………… Skjemaet sammen med kopi av registreringsskjema oppbevares i egen perm hos Elena Kamycheva

**Confirmation of participation according to the protocol “The effect of vitamin D supplementation on cardiovascular risk factors in subjects with low serum 25-hydroxyvitamin D levels” (signature and date)**

Rolf Jorde Date

Elena Kamycheva Date

Johan Svartberg Date

Ragnar Joakimsen Date

Guri Grimnes Date

Marie Kjærgaard Date

Yngve Figenschau Date

Ruth Paulssen Date

Jorid Degerstrøm Date

Arnfinn Andersen Date

Anne Sofie Furberg Date

**The effect of vitamin D supplementation on cardiovascular risk factors in subjects with low serum 25-hydroxyvitamin D levels**

Version 4, 160714 (Protocol code number: TromsøEndo-2013-1)

EUDRACT NR: 2013-003514-40. Vitamin D tilskudd og risiko for hjerte-kar sykdom

**Amendment 1, version 1, 2015-23-03**

**”Vitamin D og psoriasis”**

**Background**

Psoriasis is a chronic relapsing inflammatory immune-mediated skin disease which is associated with work productivity impairment, several comorbid conditions and possibly increased mortality (1). Over the years several studies have found a positive association between moderate to severe psoriasis and cardiovascular risk factors as well as cardio-vascular disease (CVD) (2, 3) The increased level of comorbidites among persons with psoriasis has led to the hypothesis that psoriasis is a *systemic* inflammatory condition (4); however, the exact mechanism of the associations between psoriasis, metabolic and CVD demands further research. The production of inflammatory cytokines in psoriasis has the potential to influence angiogenesis, insulin signaling, adipogenesis, lipid metabolism and immune cell trafficking, and thereby impact on other conditions like obesity, diabetes, thrombosis and atherosclerosis (5).

Interestingly, a higher prevalence of this common skin disease is seen in northern regions. In the sixth Tromsø study as many as 11% of attendees reported lifetime psoriasis (6). Patients frequently report increased severity of psoriasis in the winter, however the exact mechanisms behind the seasonal fluctuation are as of yet not established. Climate and UV(B) therapy are widely used to treat psoriasis, and under this treatment a substantial increase in serum vitamin D levels is found (7). Several smaller studies from selected patient populations have found an association between reduced serum vitamin D and psoriasis, also after adjusting for overweight, which is seen more commonly among persons with psoriasis (8). Serum vitamin D has also been inversely linked to the severity of psoriasis (8), as well as increased risk of bacterial colonization and infections (9, 10); which may play a role in the initiation or exacerbation of psoriasis (1). Some studies have found an association between upper respiratory infections and subsequent development of psoriasis, as well as psoriasis exacerbation.(11, 12) A study from the UK found an over doubled risk of psoriasis onset within one month of a respiratory tract infection with an unknown pathogen, and increased risk was also seen after infectious episodes within the last year.(11) Moreover, recent studies have suggested that the microbiome, or collective genome of microorganism that are residing in a given niche, may play an important role in immune homeostasis; where disruptions of the natural microflora, may cause immune dysfunction or autoimmunity.(13) However, studies are limited as of today.(14) Perhaps the crosstalk between microbial determinants as well as host and environmental determinant play a role in the complex puzzle that is psoriasis.

The effect of topical use of Vitamin D analogues to treat psoriasis is well established.(15)There are a few smaller ongoing trials looking into if oral vitamin D substitution can influence the severity of psoriasis (ICTRP database), but there are no randomized controlled trials to date which have investigated the effect of oral Vitamin D on the severity of psoriasis among persons who have lower serum levels. These subjects are the ones most likely to benefit from vitamin D supplementation, and accordingly those where an effect will be most easily seen.

The seventh Tromsø study will start in March 2015, and serum 25-hydroxyvitamin D (25(OH)D) (the metabolite used to evaluate a subject’s vitamin D status) will be measured in all subjects. In the study “The effect of vitamin D supplementation on cardiovascular risk factors in subjects with low serum 25-hydroxyvitamin D levels” subjects with serum 25(OH)D levels < the 7^th^ percentile will be invited to participate in a 4 months intervention study with vitamin D (100,000 IU at inclusion, thereafter 20,000 IU per week) vs placebo.

These subjects will in addition to several other research questions be asked about past or present psoriasis, and those where the diagnosis is likely and who also report symptoms within the past 12 months (See questionnaire “Hud- og Leddsykdom, Supplement 1) will be invited to this substudy where the effect on psoriasis will be evaluated.

**Hypothesis**

We believe that supplementation with vitamin D in subjects with psoriasis can influence the severity of the disease through prevention of exacerbations as well as reduction in lesion severity. We wish to investigate if this change is in part facilitated through changes in the microbial flora. Vitamin D intervention may also positively influence the level of cardiovascular risk factors including metabolic factors which are associated with psoriasis.

**Project plan**

Subjects who have accepted to participate in the study “The effect of vitamin D supplementation on cardiovascular risk factors in subjects with low serum 25-hydroxyvitamin D levels” will be given the questionnaire “Hud- og Leddsykdom” (supplement 1). If the following criteria are fulfilled; Answering yes to one or more of the following questions: 1.4, 1.5, 5.1.1, 5.1.2, 5.3.1, 5.3.2 and 5.4.1, 5.4.2; they will be given a short information about the “Vitamin D and psoriasis” substudy and if they wish to participate and/or receive more information about the study, they will be given an appointment at the Dermatology Out-patient clinic at the same day (or shortly thereafter) at the Visit 2 in the main study.

At the Dermatology Out-patient clinic the subject will be given further information by a dermatologist, and if then willing to participate, sign the informed consent form (supplement 2).

The subject will be asked to fill in two questionnaires (Dermatological Life Quality Index (DLQI) & Self-Administered Psoriasis Area Severity Indes (SAPASI)) (supplements 3 and 4) and will then undergo a short psoriasis related patient history as well as routine clinical examination by the dermatologist (supplement 5). Their present level of psoriasis will be scored by the dermatologist using the Psoriasis Area Severity Index (PASI, supplement 6), as well as the Physicians global assessment (PGA; scale of psoriasis lesions (0, clear; 1, minimal; 2, mild; 3, moderate; 4, marked; and 5, severe). In addition to self-reported medication use for psoriasis, data may be linked with the Norwegian Prescription Database to look for use of psoriasis relevant medication in the timeframe from two years before to one year after the study ends.

Routine photos will be taken of any psoriasis lesions (with patients no more undressed than wearing underwear). The persons will not be made identifiable in the pictures.

The examination will also include the joints to disclose any signs of psoriasis arthritis, and if detecting or suspecting previously unknown psoriasis arthritis, the patient will be remitted to the Rheumatology Out-patient clinic.

In the overall study persons will be swabbed for microbiome flora from the extensor side of the over extremities close to the elbow, in an area where there is no rash (see overall procedure from main study). Persons who have active psoriasis will in addition to delivering microbial swabs in the overall study also be asked to give one or more swab(s) from their active lesions taken at the Dermatology Out-patient clinic. Swabs taken in the overall study from the nasal antrum as well as throat swabs are also to the used in relation to psoriasis. Furthermore, persons who report psoriasis will be given a kit (Fecotainer) to use at first bowel movement to collect a stool sample to take along for the examination with the dermatologist or the next day. They will also be given the same Food Frequency Questionnaire as in Tromsø 7 (supplement 7). All samples will be frozen and stored for future analysis.

The anthropometric measures as well as blood samples that are planned in relation to evaluation of cardiovascular and metabolic risk markers in the overall study also apply to this substudy.

The subject will be given a form for filling in any psoriasis medication (supplement 8) as well as the DLQI and SAPASI after 8-weeks to indicate the location and severity of any psoriasis lesions.

At the end of the visit the subjects is to start the study medication (5 capsules taken at the Out –patient clinic and then 1 capsule every week).

After 6-7 weeks the subjects will be mailed the mid-evaluation form. The subjects will be called by the study nurse, and reminded to fill out the 8 week form (including medication as well as DLQI and SAPASI) regardless of if they have had any symptoms.

The second visit at the Dermatology Out-patient clinic will be after 4 months on the same day as the last visit in the main study, and the same questionnaires and examinations as in the first visit will be repeated.

**Exclusion criteria**

In addition to the inclusion criteria in the main study, subjects who during the last month have used phototherapy/ light therapy or heliotherapy as prescribed by a dermatologist, cannot be included, nor can this treatment be performed under the course of the study. Furthermore, those who have started treatment with a **new** oral or injection drug (Methotrexate, Cyclosporine, Acitretine or biological treatment (eg Humira, Remicade, Stellara ) within the last 1-2 months are excluded from entry. Furthermore, topical treatments containing vitamin D or vitamin D analogs (Including Daivobet) cannot be used during the study. If a patient uses these products regularly, replacement products which only contain local steroids will be prescribed as alternates.

**Statistical analyses**

It is anticipated that at least 11 % of the subjects in the main study will have present or past psoriasis and will be liable for inclusion in this substudy. Furthermore, we expect to find more persons with psoriasis within persons with low serum Vitamin D, anticipating approximately 80 available subjects from the 600 invited. This sample that will be recruited among subjects with psoriasis and serum 25(OH)D levels < the 7^th^ percentile in Tromsø 7, will represent a sub-cohort of a larger ‘Vitamin D and psoriasis’ cohort including psoriatics with vitamin D deficiency defined by a higher serum 25(OH)D level. Details of the study protocol for this cohort are to be determined and submitted separately. We are aiming at the inclusion of 3-400 psoriatics in total. Thereby we will achieve sufficient power to test our hypothesis.

The main endpoint will be improvement in psoriasis status after 4 months either through reduced severity of lesions, less need for treatment and fewer exacerbations.

As the variability of our outcome measures is unknown, we have generated power calculations using standardized differences (Table below). In order to have 80% power to detect a 0.5 standard deviation change in an outcome measure we will need 64 subjects in each group (intervention or placebo).

**Ethical considerations**

The study will be submitted for approval at The Regional Ethics Committee and the National Medicines Agency.

All patients with plaque psoriasis regardless of severity will be included in the study. Subjects on light- or heliotherapy within the last month will not be included in the study as this makes it difficult to evaluate the effect of Vitamin D supplementation; also it is unlikely that persons who have recently gone through this type of therapy will fall within the study inclusion criteria of low Vit D serum levels.

Subjects with worsening of psoriasis during the study necessitating initiation of local treatment with topical steroids will be allowed to use their standard treatments. If necessary, prescriptions will be given by the study doctor. Patients will be able to use light therapy after study end, and will be referred to this if necessary after study end. Also, subjects whose psoriasis cannot be controlled by local therapy will be given the possibility to start with oral therapy without exclusion from trial.

**Management**

The project leader for this substudy will be specialist in dermatology Kjersti Danielsen, MD, PhD, Department of Dermatology, University Hospital of North Norway (UNN).

**Collaborators in the study**

- Professor Rolf Jorde, Medical Department, UNN who will be responsible for the main study
- Anne-Sofie Furberg, MD PhD, UiT, The Arctic University of Norway and UNN
- Johanna Ericson Sollid, Professor in Biology, UiT, The Arctic University of Norway
- Thomas Roger Schopf, Specialist in Dermatology, PhD, UNN
- Elin Ekorness, Specialist in Dermatology, UNN
- The study will apply for a PhD candidate.

**Publication plan**

Kjersti Danielsen will be the first author of the main paper. The list of other authors on this first paper, and the list of authors on subsequent papers, will be decided by Kjersti Danielsen and Rolf Jorde.

**References:**

1. Nestle FO, Kaplan DH, Barker J. Psoriasis. The New England journal of medicine. 2009;361(5):496-509.

2. Prey S, Paul C, Bronsard V, Puzenat E, Gourraud PA, Aractingi S, et al. Cardiovascular risk factors in patients with plaque psoriasis: a systematic review of epidemiological studies. Journal of the European Academy of Dermatology and Venereology : JEADV. 2010;24 Suppl 2:23-30.

3. Armstrong EJ, Harskamp CT, Armstrong AW. Psoriasis and major adverse cardiovascular events: a systematic review and meta-analysis of observational studies. J Am Heart Assoc. 2013;2(2):e000062.

4. Davidovici BB, Sattar N, Prinz JC, Puig L, Emery P, Barker JN, et al. Psoriasis and systemic inflammatory diseases: potential mechanistic links between skin disease and co-morbid conditions. The Journal of investigative dermatology. 2010;130(7):1785-96.

5. Ucak S, Ekmekci TR, Basat O, Koslu A, Altuntas Y. Comparison of various insulin sensitivity indices in psoriatic patients and their relationship with type of psoriasis. Journal of the European Academy of Dermatology and Venereology : JEADV. 2006;20(5):517-22.

6. Danielsen K, Olsen AO, Wilsgaard T, Furberg AS. Is the prevalence of psoriasis increasing? A 30-year follow-up of a population-based cohort. The British journal of dermatology. 2013;168(6):1303-10.

7. Ala-Houhala MJ, Vahavihu K, Hasan T, Kautiainen H, Ylianttila L, Viljakainen HT, et al. Comparison of narrowband ultraviolet B exposure and oral vitamin D substitution on serum 25-hydroxyvitamin D concentration. The British journal of dermatology. 2012;167(1):160-4.

8. Gisondi P, Rossini M, Di Cesare A, Idolazzi L, Farina S, Beltrami G, et al. Vitamin D status in patients with chronic plaque psoriasis. The British journal of dermatology. 2012;166(3):505-10.

9. Ginde AA, Mansbach JM, Camargo CA, Jr. Association between serum 25-hydroxyvitamin D level and upper respiratory tract infection in the Third National Health and Nutrition Examination Survey. Archives of internal medicine. 2009;169(4):384-90.

10. Cannell JJ, Vieth R, Umhau JC, Holick MF, Grant WB, Madronich S, et al. Epidemic influenza and vitamin D. Epidemiology and infection. 2006;134(6):1129-40.

11. Huerta C, Rivero E, Rodriguez LA. Incidence and risk factors for psoriasis in the general population. Arch Dermatol. 2007;143(12):1559-65.

12. McFadden JP, Baker BS, Powles AV, Fry L. Psoriasis and streptococci: the natural selection of psoriasis revisited. The British journal of dermatology. 2009;160(5):929-37.

13. Fung I, Garrett JP, Shahane A, Kwan M. Do bugs control our fate? The influence of the microbiome on autoimmunity. Curr Allergy Asthma Rep. 2012;12(6):511-9.

14. Trivedi B. Microbiome: The surface brigade. Nature. 2012;492(7429):S60-1.

15. Mason A, Mason J, Cork M, Hancock H, Dooley G. Topical treatments for chronic plaque psoriasis: an abridged Cochrane systematic review. Journal of the American Academy of Dermatology. 2013;69(5):799-807.

**The effect of vitamin D supplementation on cardiovascular risk factors in subjects with low serum 25-hydroxyvitamin D levels (D-COR)**

EUDRACT NR: 2013-003514-40. Vitamin D tilskudd og risiko for hjerte-kar sykdom

Amendment 2. Effect of vitamin D supplementation on bone mass density (BMD) in the D-COR study (08.07.2015)

In the D-COR study subjects with low serum 25-hydroxyvitamin D 25(OH)D) will be included in a 4 month intervention with vitamin D (20.000 IU per week) versus placebo.

Low serum 25(OH)D levels are associated with reduced BMD (1). For subjects with very low serum 25(OH)D levels this may in addition to osteoporosis also represent various degrees of osteomalacia (2).

Osteomalacia is defined as mineralization defect of bone matrix, leading to low mineral content of the skeleton. The main cause of osteomalacia is vitamin D deficiency. The clinical features of full blown osteomalacia include musculoskeletal pain and muscle weakness. However, in its mild and early stages, osteomalacia may only present as osteopenia and osteoporosis.

In response to treatment, reduced BMD due to osteomalacia respond very quickly with the main increase within the first few months ( 3). The increase depends on degree of osteomalacia and mean increases of 27 % for the lumbal spine and 31 % for the femoral neck have been described (4).

It is unlikely that our subjects will have severe osteomalacia and subjects with symptoms like muscle pain and muscle weakness will not be included in the present study. However, decreased mineralization due to vitamin D deficiency may be a feature in some of our subjects.

In the ongoing discussion on need for vitamin D supplementation and threshold for sufficient level of 25(OH)D, effect (or no effect) on BMD in our study will be very important.

If conservatively estimating a 2.2 % increase in BMD similar to that seen with Denosunab or Zoledronate during the first 4 month of treatment (5), a mean BMD of the lumbal spine of 0.90 g/cm^2^, a SD of 0.07 g/cm^2^ (6), and wanting a P value < 0.05 and power 0.8, we would need to include 340 subjects. Accordingly, we do have enough power in the present study where we aim to include 600 subjects

In the D-COR study we will therefore include BMD of total body, hip and spine measured with DEXA before and after the intervention.

**Follow-up**

Subjects with BMD T-score < -3.5 at the spine or hip measured with DEXA before the intervention will be offered treatment with bisphosphonate and can continue in the study.

After the intervention all subjects with BMD T-score < - 2.5 at the spine or the hip will individually be advised on treatment and followed-up according to standard clinical routines.

References

1. [Kuchuk NO](http://www.ncbi.nlm.nih.gov/pubmed/?term=Kuchuk%20NO%5BAuthor%5D&cauthor=true&cauthor_uid=19049341), [van Schoor NM](http://www.ncbi.nlm.nih.gov/pubmed/?term=van%20Schoor%20NM%5BAuthor%5D&cauthor=true&cauthor_uid=19049341), [Pluijm SM](http://www.ncbi.nlm.nih.gov/pubmed/?term=Pluijm%20SM%5BAuthor%5D&cauthor=true&cauthor_uid=19049341), [Chines A](http://www.ncbi.nlm.nih.gov/pubmed/?term=Chines%20A%5BAuthor%5D&cauthor=true&cauthor_uid=19049341), [Lips P](http://www.ncbi.nlm.nih.gov/pubmed/?term=Lips%20P%5BAuthor%5D&cauthor=true&cauthor_uid=19049341). Vitamin D status, parathyroid function, bone turnover, and BMD in postmenopausal women with osteoporosis: global perspective. [J Bone Miner Res.](http://www.ncbi.nlm.nih.gov/pubmed/19049341) 2009 Apr;24(4):693-701. doi: 10.1359/jbmr.081209.
2. [Reid IR](http://www.ncbi.nlm.nih.gov/pubmed/?term=Reid%20IR%5BAuthor%5D&cauthor=true&cauthor_uid=24119980), [Bolland MJ](http://www.ncbi.nlm.nih.gov/pubmed/?term=Bolland%20MJ%5BAuthor%5D&cauthor=true&cauthor_uid=24119980), [Grey A](http://www.ncbi.nlm.nih.gov/pubmed/?term=Grey%20A%5BAuthor%5D&cauthor=true&cauthor_uid=24119980). Effects of vitamin D supplements on bone mineral density: a systematic review and meta-analysis. [Lancet.](http://www.ncbi.nlm.nih.gov/pubmed/?term=Effects+of+vitamin+D+supplements+on+bone+mineral+density%3A+a+systematic+review+and+meta-analysis.) 2014 Jan 11;383(9912):146-55. doi: 10.1016/S0140-6736(13)61647-5. Epub 2013 Oct 11.
3. [Bhambri R](http://www.ncbi.nlm.nih.gov/pubmed/?term=Bhambri%20R%5BAuthor%5D&cauthor=true&cauthor_uid=16731441), [Naik V](http://www.ncbi.nlm.nih.gov/pubmed/?term=Naik%20V%5BAuthor%5D&cauthor=true&cauthor_uid=16731441), [Malhotra N](http://www.ncbi.nlm.nih.gov/pubmed/?term=Malhotra%20N%5BAuthor%5D&cauthor=true&cauthor_uid=16731441), [Taneja S](http://www.ncbi.nlm.nih.gov/pubmed/?term=Taneja%20S%5BAuthor%5D&cauthor=true&cauthor_uid=16731441), [Rastogi S](http://www.ncbi.nlm.nih.gov/pubmed/?term=Rastogi%20S%5BAuthor%5D&cauthor=true&cauthor_uid=16731441), [Ravishanker U](http://www.ncbi.nlm.nih.gov/pubmed/?term=Ravishanker%20U%5BAuthor%5D&cauthor=true&cauthor_uid=16731441), [Mithal A](http://www.ncbi.nlm.nih.gov/pubmed/?term=Mithal%20A%5BAuthor%5D&cauthor=true&cauthor_uid=16731441). Changes in bone mineral density following treatment of osteomalacia. [J Clin Densitom.](http://www.ncbi.nlm.nih.gov/pubmed/16731441) 2006 Jan-Mar;9(1):120-7. Epub 2006 Mar 27.
4. [Basha B](http://www.ncbi.nlm.nih.gov/pubmed/?term=Basha%20B%5BAuthor%5D&cauthor=true&cauthor_uid=11014722), [Rao DS](http://www.ncbi.nlm.nih.gov/pubmed/?term=Rao%20DS%5BAuthor%5D&cauthor=true&cauthor_uid=11014722), [Han ZH](http://www.ncbi.nlm.nih.gov/pubmed/?term=Han%20ZH%5BAuthor%5D&cauthor=true&cauthor_uid=11014722), [Parfitt AM](http://www.ncbi.nlm.nih.gov/pubmed/?term=Parfitt%20AM%5BAuthor%5D&cauthor=true&cauthor_uid=11014722). Osteomalacia due to vitamin D depletion: a neglected consequence of intestinal malabsorption. [Am J Med.](http://www.ncbi.nlm.nih.gov/pubmed/?term=Osteomalacia+due+to+vitamin+D+depletion%3A+a+neglected+consequence+of+intestinal+malabsorption) 2000 Mar;108(4):296-300.
5. [Reid IR](http://www.ncbi.nlm.nih.gov/pubmed/?term=Reid%20IR%5BAuthor%5D&cauthor=true&cauthor_uid=25963272). Short-term and long-term effects of osteoporosis therapies. [Nat Rev Endocrinol.](http://www.ncbi.nlm.nih.gov/pubmed/25963272) 2015 May 12. doi: 10.1038/nrendo.2015.71. [Epub ahead of print]
6. [Grimnes G](http://www.ncbi.nlm.nih.gov/pubmed/?term=Grimnes%20G%5BAuthor%5D&cauthor=true&cauthor_uid=21909730), [Joakimsen R](http://www.ncbi.nlm.nih.gov/pubmed/?term=Joakimsen%20R%5BAuthor%5D&cauthor=true&cauthor_uid=21909730), [Figenschau Y](http://www.ncbi.nlm.nih.gov/pubmed/?term=Figenschau%20Y%5BAuthor%5D&cauthor=true&cauthor_uid=21909730), [Torjesen PA](http://www.ncbi.nlm.nih.gov/pubmed/?term=Torjesen%20PA%5BAuthor%5D&cauthor=true&cauthor_uid=21909730), [Almås B](http://www.ncbi.nlm.nih.gov/pubmed/?term=Alm%C3%A5s%20B%5BAuthor%5D&cauthor=true&cauthor_uid=21909730), [Jorde R](http://www.ncbi.nlm.nih.gov/pubmed/?term=Jorde%20R%5BAuthor%5D&cauthor=true&cauthor_uid=21909730). The effect of high-dose vitamin D on bone mineral density and bone turnover markers in postmenopausal women with low bone mass--a randomized controlled 1-year trial. [Osteoporos Int.](http://www.ncbi.nlm.nih.gov/pubmed/21909730) 2012 Jan;23(1):201-11. doi: 10.1007/s00198-011-1752-5. Epub 2011 Sep 10.

**The effect of vitamin D supplementation on cardiovascular risk factors in subjects with low serum 25-hydroxyvitamin D levels**

Version 5, 221215 (Protocol code number: TromsøEndo-2013-1)

EUDRACT NR: 2013-003514-40. Vitamin D tilskudd og risiko for hjerte-kar sykdom

**Amendment 3, version 1, 2015-22-12**

**”Vitamin D and adipose tissue biopsies”**

(Norwegian short title «Vitamin D og fettbiopsier»)

Rolf Jorde, Yvonne Pasing, Einar Jensen, Jette Jakobsen and Ieva Zostautiene

Tromsø Endocrine Research Group, Institute of Clinical Medicine and Institute of Pharmacology, UiT – the Artic University of Norway; and National Food Institute, Technical University of Denmark, Søborg, Denmark

**Content**

| English and Norwegian summaries | 2 |
| --- | --- |
| Background | 2 |
| Storage of vitamin D in adipose tissue | 3 |
| Effects on adipose tissue proteome | 4 |
| Subjects and recruitment procedure | 5 |
| The biopsy procedure | 6 |
| Analyses | 7 |
| Power calculation | 7 |
| Statistical analyses | 7 |
| Data handling | 7 |
| Ethics | 7 |
| Project group | 8 |
| Progress plan | 8 |
| Budget | 8 |
| References | 8 |

**Summary**

We have previously performed two studies with fat biopsies, “Duration of vitamin D stores after prolonged vitamin D substitution” (REK nr 2012/756; EUDRACTNR – 2007-002167-27) and “The GLOBAL vitamin D study. A genomic, transcriptomic, proteomic and metabolomic approach to the effects of vitamin D in adipose tissue and peripheral blood” (REK nr 2012/766; EUDRACT NR: 2012-002082-35).

In these fat biopsies we have found substantial storage of vitamin D after 3 – 5 years with vitamin D supplementation as well as significant over-all effects on the proteomic profile. In the present study we will investigate if supplementation with vitamin D for 4 months (similar to the effect of sunshine during the summer) will result in significant vitamin D storage, and also elaborate on the proteomic effects by using a more targeted approach. Therefore, eligible subjects who are to participate in the ongoing study ”The effect of vitamin D supplementation on cardiovascular risk factors in subjects with low serum 25-hydroxyvitamin D levels” will be invited to the present sub-study with fat biopsies taken before and after the four month supplementation with vitamin D 20.000 IU per week or placebo. The biopsies will be analyzed for vitamin D content as well as for targeted protein profiles. Forty subjects will be included.

**Norsk sammendrag**

Vi har tidligere gjennomført to studier med fettbiopsier, «Varighet av vitamin D lagre etter langvarig vitamin D tilskudd» (REK nr 2012/756; EUDRACTNR – 2007-002167-27) og «Den GLOBALE vitamin D studien» (REK nr 2012/766; EUDRACT NR: 2012-002082-35).

I disse fettbiopsiene har vi påvist betydelig lagring av vitamin D, samt at vitamin D tilskudd påvirker protein profilen. I aktuelle studie vil vi undersøke om tilskudd med vitamin D over 4 måneder (tilsvarende sol effekten om sommeren) vil resultere i vitamin D lagring av betydning, samt å se mer detaljert på protein effekten med mer sentrerte og målrettede analyser. Vi vil derfor invitere personer som skal delta i studien «Vitamin D tilskudd og risiko for hjerte-kar sykdom», og som fyller enkelte tilleggskriterier, til å delta i denne sub-studien der det vil bli tatt fettbiopsier før og etter 4 måneder med vitamin D tilskudd (20.000 IU per uke) eller placebo. Biopsiene vil bli analysert for vitamin D innhold og spesifikke protein profiler. Førti personer vil bli inkludert.

**Background**

The main source of vitamin D is solar UV-radiation, and except for fatty fish there are few dietary sources. Vitamin D is hydroxylated in the liver to 25(OH)D which is used as a marker of a subject’s vitamin D status. 25(OH)D serves as a substrate for 1-α-hydroxylase in the kidneys, which under tight regulation by parathyroid hormone (PTH) forms the active form of the vitamin, 1,25-dihydroxyvitamin D (1,25(OH)_2_ D). The enzyme 1-α-hydroxylase has also been found in extra-renal tissues, and activation of vitamin D can therefore occur locally. What regulates this local activation is not known. The active form of vitamin D binds to the nuclear receptor VDR. This receptor is found in tissues throughout the body (1). The serum levels of 25(OH)D and 1,25(OH)_2_D and the function of the VDR are also determined by genetic factors (2, 3).

Low serum 25(OH)D levels have been associated with a number of diseases (4) as well as with risk factors/markers like blood pressure (BP) (5) and lipids (6). However, RCTs and their meta-analyses have not shown conclusive results (7-9).

Therefore, an “umbrella-review” with meta-analysis of the meta-analyses had to conclude that: “Despite a few hundred systematic reviews and meta-analyses, highly convincing evidence of a clear role of vitamin D does not exist for any outcome, but associations with a selection of outcomes are probable”; and…. “Further studies and better designed trials are needed to draw firmer conclusions” (10).

However, most of these RCTs have not included subjects who were clearly vitamin D deficient. Thus, in the most recent meta-analysis on BP using individual person data only one out of 10 subjects had a serum 25(OH)D level < 25 nmol/L (20), and in only one out of 15 studies on glucose metabolism had the subjects at baseline a mean serum 25(OH)D level < 30 nmol/L (11). The chance of a type 2 error is obvious.

In 2015 we therefore started the study “The effect of vitamin D supplementation on cardiovascular risk factors in subjects with low serum 25-hydroxyvitamin D levels” where subjects with low serum 25(OH)D levels detected in the 7^th^ Tromsø study are included. Six hundred subjects will be randomized to vitamin D (20.000 IU per week) versus placebo for four months with effects on blood pressure, lipids and glucose metabolism as primary endpoints, and effects on measures of bone formation, arterial function, muscle strength, mood, bacterial flora, and cognitive function as secondary endpoints.

In the present sub-study we want to include fat biopsies taken before start and at the end of the four months’ intervention in 40 subjects to evaluate:

1. Vitamin D content (storage) in adipose tissue and
2. Effects on the proteome in adipose tissue

**Storage of vitamin D in adipose tissue**

Ingested or skin-produced vitamin D is either hydroxylated to 25(OH)D in the liver, degraded to other metabolites and excreted, or stored in fat or other tissues.

Vitamin D is consistently measureable in fat tissue. In a study by Blum et al. (12) levels of vitamin D in adipose tissue was ~ 100 nmol/kg, similar to that previously reported by Lawson et al. (13). Of note was the wide range of vitamin D content from 48 to 187 nmol/kg, and also that there was a correlation with the serum level of vitamin D (8). Furthermore, Blum et al. made the observation that a subject with remarkably high serum 25(OH)D and vitamin D levels had a low fat vitamin D level, presumably because there was a very short time period between a heavy sun exposure and the fat biopsy (12). Accordingly, fat storage may not be a rapid process. The wide range in vitamin D content in fat tissue has been confirmed in a study by Pramyothin et al. on morbidly obese subjects where the range was from 4 to 2.470 ng/g. (14). In line with this, the capacity of fat to store vitamin D may be considerable as demonstrated in an experiment by Lawson et al. where rats were exposed to prolonged UVB irradiation and the fat vitamin D content increased 10 fold (13). However, the importance of vitamin D storage in fat is by some considered to be of minor importance (15).

How the storage of vitamin D in adipose tissue is regulated is uncertain. In a recent publication by Drincic et al. (16) it was argued that the vitamin D in fat tissue, vitamin D in serum and 25(OH)D in serum were in equilibrium with each other, with simple passive diffusion between serum and fat cells for vitamin D. They also estimated that the relative affinities for vitamin D between DBP (the main binding protein in serum) and vitamin D in body fat depots to be approximately 1:12. In this model the 25-hydroxylation of vitamin D to 25(OH)D would be a competitive pathway, pulling vitamin D out of the fat by hepatic uptake of vitamin D from the circulation.

However, the storage capacity for vitamin D in fat tissue in humans is not determined. Nor is it known if the storage is an active or passive process. This could operate differently in subjects according to their vitamin D status, and in vitamin D deficiency the 25 and 1- α-hydroxylation of vitamin D are more efficient and the 24-hydroxylation (degradation) tuned down. Accordingly, even a modest storage of vitamin D in fat tissue could under certain circumstances be of vital importance.

We have previously in collaboration with Senior Scientist Jette Jakobsen, National Food Institute, Technical University of Denmark, documented such storage in subjects after prolonged high-dose vitamin D supplementation (17). Thus, in 29 subjects with impaired glucose tolerance who had participated in a randomized controlled trial with vitamin D3 20 000 IU (500 μg) per week vs placebo for 3-5 years, abdominal subcutaneous fat tissue was obtained by needle biopsy for the measurements of vitamin D3 and 25-hydroxyvitamin D3 (25(OH)D3). In the subjects given vitamin D3, the median concentration of fat vitamin D3 was 209 ng/g, and correspondingly in the placebo group 32 ng/g. If assuming an equal amount of vitamin D3 stored in all adipose tissue in the body, the median body store was 66 00 ug vitamin D3 in those given vitamin D3. This amount of vitamin D stored is equivalent to 660 spoonful of cod liver oil (17).

However, the subjects in that study had been given vitamin D for 3-5 years and the extent of storage of vitamin D after short term surplus, like during summer sun exposure (~ 4 months), is not known. This is of importance to explain why the serum 25(OH)D levels do not fall to extremely low levels during the Nordic winter. In this regard it should be considered that the half-life of 25(OH)D is close to 4 weeks (1), and that the intake of fatty fish and supplements is very low in the majority of the population.

To demonstrate the capacity of storage from such short term vitamin D surplus, we will take fat biopsies from 40 subjects before and after the 4 months vitamin D supplementation.

**Effects on adipose tissue proteome**

Proteomics is the large-scale study of [proteins](http://en.wikipedia.org/wiki/Protein), particularly their [structures](http://en.wikipedia.org/wiki/Protein_structure) and [functions](http://en.wikipedia.org/wiki/Functional_genomics) (18). Proteins are vital parts of living organisms, as they are the main components of the physiological [metabolic pathways](http://en.wikipedia.org/wiki/Metabolic_pathways) of [cells](http://en.wikipedia.org/wiki/Biological_cell). The proteome can be defined as the proteins present in one sample (organism, tissue, body fluid, cell culture) at a certain point in time (19). Unlike the genome, which is relatively static, the proteome changes constantly in response to tens of thousands of intra- and extracellular environmental signals. The proteome varies with health or disease, the nature of each tissue, the stage of cell development, and effects of drug treatments. In addition to the constant changes, the share number of proteins makes the study of the proteome challenging. Thus, the ~ 30 000 genes in the human genome can code for at least ten times as many proteins; in extreme cases a single gene alone can code for over 1000 proteins.

Owing to the performance of mass spectrometry technology, current high-throughput proteomic analysis allows for the identification of a high number of these proteins. This has been used to discover biomarkers of disease, particularly in oncology (20), and has also proved to be helpful in drug discovery and development (21). So far there are no reports on the effect of vitamin D substitution on the proteome.

In the study “The GLOBAL vitamin D study. A genomic, transcriptomic, proteomic and metabolomic approach to the effects of vitamin D in adipose tissue and peripheral blood” we included 19 subjects and randomized to vitamin D (40.000 per week) versus placebo for 2 months. Analysis of the adipose tissue samples showed clear effects on the proteome in an untargeted, broad approach (“all” protein analyzed without particular focus on specific groups or pathways). The placebo and vitamin D groups could be separated using multivariate data analysis on the quantitative proteomic data obtained. The regulated proteins that were found to be affected by (regulated by) vitamin D, were involved in biological processes like protein complex assembly and biogenesis, regulation of liquid surface tension, protein folding, mRNA splicing and processing, oxidative phosphorylation, generation of precursor metabolites and energy and carbohydrate metabolic process (unpublished data).

In the present sub-study we will therefore do targeted analyses of the proteome where only the pathways and functions listed above will be analyzed. Doing this targeted analysis will hopefully not only confirm the initial findings, but do so with a much higher statistical power as the number of proteins analyzed will be a fraction of those evaluated in the GLOBAL study.

**Subjects and recruitment procedure**

The subjects (men and women) in the main study are invited based on serum 25(OH)D measurement < 10^th^ percentile in the ongoing 7^th^ Tromsø study.

The exclusion criteria for the main study are

- subjects allergic to nuts (the study capsules contain peanut oil)
- subjects with primary hyperparathyroidism
- granulomatous diseases (sarcoidosis, tuberculosis, Wegner’s granulomatosis)
- reduced kidney function (creatinine > 130 μmol/L in males and 120 μmol/L in females)
- systolic BP > 174 mmHg, diastolic BP > 104 mmHg,
- diabetes (fasting blood glucose > 6.9 mmol/L and/or HbA_1c_ > 6.5 %)
- renal stones the last five years
- subjects who use solarium on a regular basis (more than twice a month on average)
- subjects who plan holiday(s) in tropical areas (or Mediterranean during the summer months) for more than two weeks
- subjects with BDI score higher than 29
- subjects with clinical signs of proximal myopathy (problems with standing up from chair or walking stairs)
- subjects seriously ill and unfit for participation in a clinical study (as judged by one of the study doctors)
- subjects using vitamin D supplements exceeding 800 IU per day or active vitamin D drugs (Rocaltrol or Etalpha)

Since the proteome is sensitive to changes in general, the following additional exclusion criteria apply to this sub-study:

- smoking
- men > 70 years
- women < 60 years (to avoid influence of menstrual cycle)
- subjects who plan to lose weight
- subjects with change of medication last 4 weeks
- BMI > 32 kg/m^2^ or BMI < 21 kg/m^2^

To avoid hematoma after the biopsy, subjects who use anti-coagulants (except Albyl E < 75 mg) will not be included.

All subjects who respond to invitation in the main study are screened by telephone before they come to the first visit. At that screening interview additional questions on smoking, weight loss, medication and BMI will be added. They will also at the telephone interview be informed about the sub-study (and that they may participate in the main study without participating in the sub-study). If they at the first visit consent to participate in the sub-study, a separate informed consent form has to be signed. The biopsy will be performed at a separate visit after an overnight fast.

**The biopsy procedure**

The adipose tissue biopsy will be taken after local anesthesia as described by Mutch et al. (22), standardized in accordance with previous work performed within the European Framework 5 project NUGENOB (http://www.nugenob.com/): “Superficial subcutaneous adipose tissue samples of 1–2 cm^3^ (corresponding to 1–2 g) are obtained from the periumbilical area, under local anesthesia (1% xylocaine). A half circular small dermal injection (intracutaneous) is made, and 2 mL of a local anesthetic agent injected. After 5 min, the skin is sterilized again. A needle (BD Microlance Hypodermic Needle, 16 G, 40 mm, regular bevel, part no. 300637; Becton Dickinson France SAS, Le Pont-De-Claix, France) is then adapted to a 20-mL syringe and the piston compressed. Approximately one-third of the length of the needle is inserted into the subcutaneous fat, and the needle piston released maximally until it locked by a stopper, thereby creating a vacuum. Tissue resistance is created by gripping the abdominal wall with one hand while the other hand rotates the needle throughout the tissue in an up-down motion. Once the tissue is aspirated by the syringe, the needle is withdrawn, and the piston removed; adipose tissue samples are washed in physiologic saline, placed immediately in liquid nitrogen, and stored at -80^o^C until analysis. Needle-aspirated biopsies that appear bloody are not to be used.”

**Analyses**

The fat biopsies will be analyzed for vitamin D by Senior Scientist Jette Jakobsen, Division of Food Chemistry, Technical University of Denmark, Søborg (allan).

The proteomic profile will be analyzed with mass spectrometry technology at UiT – the Arctic University of Norway

**Power calculation**

The power calculation is based on storage of vitamin D in adipose tissue. In our previous study we have found the concentration of vitamin D in adipose tissue to be 32 + 16 ng/g. If considering a 50 % increase (16 ng/g) to be of clinical significance and if wanting a power of 0.8 and a P value < 0.05, then 30 subjects need to be included. To make sure that at least 30 subjects also will have the second biopsy, we will include 40 subjects in the trial.

As for the proteomic part of the study, a separate power calculation is not performed. However, significant effects were seen with a global approach in only 19 subjects, and given that we now will do a targeted approach, we will have more than enough power when the number of subjects is increased to 30.

**Statistical analyses**

Storage of vitamin D in adipose tissue biopsies in the two groups will be analyzed with regression analyses adjusting for baseline level, sex, age, season (mid-month) and BMI. Changes in the proteome will be analyzed with the Waters software MarkerLynx™XS Application Manager. This software mainly applies **Multivariate Statistical Analysis and also Visualization to identify relevant biomarkers.**

**Data handling**

Data handling will be as in the main study.

**Ethics**

The project will be submitted to the Regional Ethics Committee and the Norwegian Medicines Agency. The subjects will be given a gift card of 500 NKR for each of the fat biopsies (a total of 1000 NKR).

**Project group**

- Professor Rolf Jorde, Department of Clinical Medicine, UiT, will be the project leader
- PhD student Ieva Zostautiene, Medical Department, University hospital of North Norway will perform the fat biopsies
- Professor Einar Jensen, UiT and Yvonne Pasing, research fellow, MSc, will perform the proteomic analyses
- Senior Scientist Jette Jakobsen, National Food Institute, Technical University of

Denmark, Søborg, Denmark will analyze vitamin D in adipose tissue

**Progress plan**

To avoid the influence of vitamin D production in the skin, the recruitment period will be from September 1 till December 1, 2016; and accordingly, the last subject will come to the last visit no later than April 1, 2017.

**Budget**

The project will be financed by funds available to The Tromsø Endocrine Research Group

**References**

1. [DeLuca HF](http://www.ncbi.nlm.nih.gov/pubmed?term=DeLuca%20HF%5BAuthor%5D&cauthor=true&cauthor_uid=15585789). Overview of general physiologic features and functions of vitamin D. Am J Clin Nutr 2004;80(6 Suppl):1689S-96S
2. [Wang TJ](http://www.ncbi.nlm.nih.gov/pubmed?term=%22Wang%20TJ%22%5BAuthor%5D), et al. Common genetic determinants of vitamin D insufficiency: a genome-wide association study. [Lancet](javascript:AL_get(this,%20'jour',%20'Lancet.');) 2010; 376: 180-8
3. Raimondi S, et al. [Review and meta-analysis on vitamin D receptor polymorphisms and cancer risk.](http://www.ncbi.nlm.nih.gov/pubmed/19403841) Carcinogenesis 2009; 30: 1170-80
4. Holick MF. Vitamin D deficiency. [N Engl J Med](javascript:AL_get(this,%20'jour',%20'N%20Engl%20J%20Med.');) 2007; 357: 266-81
5. Jorde R et al. Serum 25-hydroxyvitamin D levels are strongly related to systolic blood pressure but do not predict future hypertension. Hypertension 2010; 55: 792-8
6. Jorde R & [Grimnes G](http://www.ncbi.nlm.nih.gov/pubmed?term=Grimnes%20G%5BAuthor%5D&cauthor=true&cauthor_uid=21640757). Vitamin D and metabolic health with special reference to the effect of vitamin D on serum lipids. [Prog Lipid Res](http://www.ncbi.nlm.nih.gov/pubmed/21640757) 2011; 50: 303-12
7. [Bolland MJ](http://www.ncbi.nlm.nih.gov/pubmed?term=Bolland%20MJ%5BAuthor%5D&cauthor=true&cauthor_uid=24703049) et al. The effect of vitamin D supplementation on skeletal, vascular, or cancer outcomes: a trial sequential meta-analysis. Lancet Diabetes Endocrinol 2014;2:307-20
8. [Beveridge LA](http://www.ncbi.nlm.nih.gov/pubmed/?term=Beveridge%20LA%5BAuthor%5D&cauthor=true&cauthor_uid=25775274), et al. Effect of Vitamin D Supplementation on Blood Pressure: A Systematic Review and Meta-analysis Incorporating Individual Patient Data. JAMA Intern Med. 2015 [Epub ahead of print]
9. [George PS](http://www.ncbi.nlm.nih.gov/pubmed?term=George%20PS%5BAuthor%5D&cauthor=true&cauthor_uid=22486204), et al. Effect of vitamin D supplementation on glycaemic control and insulin resistance: a systematic review and meta-analysis. [Diabet Med](http://www.ncbi.nlm.nih.gov/pubmed/22486204) 2012; 29: e142-50
10. [Theodoratou E](http://www.ncbi.nlm.nih.gov/pubmed?term=Theodoratou%20E%5BAuthor%5D&cauthor=true&cauthor_uid=24690624) et al. Vitamin D and multiple health outcomes: umbrella review of systematic reviews and meta-analyses of observational studies and randomised trials. BMJ 2014;348:g2035
11. [Jorde R](http://www.ncbi.nlm.nih.gov/pubmed/?term=Jorde%20R%5BAuthor%5D&cauthor=true&cauthor_uid=25636723), [Grimnes G](http://www.ncbi.nlm.nih.gov/pubmed/?term=Grimnes%20G%5BAuthor%5D&cauthor=true&cauthor_uid=25636723). Vitamin D and health: The need for more randomized controlled trials. J Steroid Biochem Mol Biol. 2015 Apr;148:269-74
12. [Blum M](http://www.ncbi.nlm.nih.gov/pubmed?term=%22Blum%20M%22%5BAuthor%5D), [Dolnikowski G](http://www.ncbi.nlm.nih.gov/pubmed?term=%22Dolnikowski%20G%22%5BAuthor%5D), [Seyoum E](http://www.ncbi.nlm.nih.gov/pubmed?term=%22Seyoum%20E%22%5BAuthor%5D), [Harris SS](http://www.ncbi.nlm.nih.gov/pubmed?term=%22Harris%20SS%22%5BAuthor%5D), [Booth SL](http://www.ncbi.nlm.nih.gov/pubmed?term=%22Booth%20SL%22%5BAuthor%5D), [Peterson J](http://www.ncbi.nlm.nih.gov/pubmed?term=%22Peterson%20J%22%5BAuthor%5D), [Saltzman E](http://www.ncbi.nlm.nih.gov/pubmed?term=%22Saltzman%20E%22%5BAuthor%5D), [Dawson-Hughes B](http://www.ncbi.nlm.nih.gov/pubmed?term=%22Dawson-Hughes%20B%22%5BAuthor%5D). Vitamin D(3) in fat tissue. [Endocrine.](http://www.ncbi.nlm.nih.gov/pubmed/18338271) 2008 Feb;33(1):90-4.
13. [Lawson DE](http://www.ncbi.nlm.nih.gov/pubmed?term=%22Lawson%20DE%22%5BAuthor%5D), [Douglas J](http://www.ncbi.nlm.nih.gov/pubmed?term=%22Douglas%20J%22%5BAuthor%5D), [Lean M](http://www.ncbi.nlm.nih.gov/pubmed?term=%22Lean%20M%22%5BAuthor%5D), [Sedrani S](http://www.ncbi.nlm.nih.gov/pubmed?term=%22Sedrani%20S%22%5BAuthor%5D). Estimation of vitamin D3 and 25-hydroxyvitamin D3 in muscle and adipose tissue of rats and man. [Clin Chim Acta.](http://www.ncbi.nlm.nih.gov/pubmed?term=lawson%20de%20and%20lean%20m) 1986 Jun 15;157(2):175-81.
14. [Pramyothin P](http://www.ncbi.nlm.nih.gov/pubmed?term=%22Pramyothin%20P%22%5BAuthor%5D), [Biancuzzo RM](http://www.ncbi.nlm.nih.gov/pubmed?term=%22Biancuzzo%20RM%22%5BAuthor%5D), [Lu Z](http://www.ncbi.nlm.nih.gov/pubmed?term=%22Lu%20Z%22%5BAuthor%5D), [Hess DT](http://www.ncbi.nlm.nih.gov/pubmed?term=%22Hess%20DT%22%5BAuthor%5D), [Apovian CM](http://www.ncbi.nlm.nih.gov/pubmed?term=%22Apovian%20CM%22%5BAuthor%5D), [Holick MF](http://www.ncbi.nlm.nih.gov/pubmed?term=%22Holick%20MF%22%5BAuthor%5D). Vitamin D in adipose tissue and serum 25-hydroxyvitamin D after roux-en-Y gastric bypass. [Obesity (Silver Spring).](http://www.ncbi.nlm.nih.gov/pubmed/21701564) 2011 Nov;19(11):2228-34. doi: 10.1038/oby.2011.170.
15. [Heaney RP](http://www.ncbi.nlm.nih.gov/pubmed?term=%22Heaney%20RP%22%5BAuthor%5D), [Recker RR](http://www.ncbi.nlm.nih.gov/pubmed?term=%22Recker%20RR%22%5BAuthor%5D), [Grote J](http://www.ncbi.nlm.nih.gov/pubmed?term=%22Grote%20J%22%5BAuthor%5D), [Horst RL](http://www.ncbi.nlm.nih.gov/pubmed?term=%22Horst%20RL%22%5BAuthor%5D), [Armas LA](http://www.ncbi.nlm.nih.gov/pubmed?term=%22Armas%20LA%22%5BAuthor%5D). Vitamin D(3) is more potent than vitamin D(2) in humans. [J Clin Endocrinol Metab.](http://www.ncbi.nlm.nih.gov/pubmed?term=heaney%20rp%20and%20grote%20j) 2011 Mar;96(3):E447-52.
16. [Drincic AT](http://www.ncbi.nlm.nih.gov/pubmed?term=%22Drincic%20AT%22%5BAuthor%5D), [Armas LA](http://www.ncbi.nlm.nih.gov/pubmed?term=%22Armas%20LA%22%5BAuthor%5D), [Van Diest EE](http://www.ncbi.nlm.nih.gov/pubmed?term=%22Van%20Diest%20EE%22%5BAuthor%5D), [Heaney RP](http://www.ncbi.nlm.nih.gov/pubmed?term=%22Heaney%20RP%22%5BAuthor%5D). Volumetric Dilution, Rather Than Sequestration Best Explains the Low Vitamin D Status of Obesity. [Obesity (Silver Spring).](http://www.ncbi.nlm.nih.gov/pubmed?term=drincic%20at) 2012 Jan 19. doi: 10.1038/oby.2011.404.
17. [Didriksen A](http://www.ncbi.nlm.nih.gov/pubmed/?term=Didriksen%20A%5BAuthor%5D&cauthor=true&cauthor_uid=25661743), [Burild A](http://www.ncbi.nlm.nih.gov/pubmed/?term=Burild%20A%5BAuthor%5D&cauthor=true&cauthor_uid=25661743), [Jakobsen J](http://www.ncbi.nlm.nih.gov/pubmed/?term=Jakobsen%20J%5BAuthor%5D&cauthor=true&cauthor_uid=25661743), [Fuskevåg OM](http://www.ncbi.nlm.nih.gov/pubmed/?term=Fuskev%C3%A5g%20OM%5BAuthor%5D&cauthor=true&cauthor_uid=25661743), [Jorde R](http://www.ncbi.nlm.nih.gov/pubmed/?term=Jorde%20R%5BAuthor%5D&cauthor=true&cauthor_uid=25661743). Vitamin D3 increases in abdominal subcutaneous fat tissue after supplementation with vitamin D3. [Eur J Endocrinol.](http://www.ncbi.nlm.nih.gov/pubmed/25661743) 2015 Mar;172(3):235-41. doi: 10.1530/EJE-14-0870.
18. [Anderson NL](http://www.ncbi.nlm.nih.gov/pubmed?term=%22Anderson%20NL%22%5BAuthor%5D), [Anderson NG](http://www.ncbi.nlm.nih.gov/pubmed?term=%22Anderson%20NG%22%5BAuthor%5D). Proteome and proteomics: new technologies, new concepts, and new words. [Electrophoresis.](http://www.ncbi.nlm.nih.gov/pubmed?term=Proteome%20and%20proteomics%3A%20new%20technologies%2C%20new%20concepts%2C%20and%20new%20words##) 1998 Aug;19(11):1853-61.
19. [Wilkins MR](http://www.ncbi.nlm.nih.gov/pubmed?term=%22Wilkins%20MR%22%5BAuthor%5D), [Pasquali C](http://www.ncbi.nlm.nih.gov/pubmed?term=%22Pasquali%20C%22%5BAuthor%5D), [Appel RD](http://www.ncbi.nlm.nih.gov/pubmed?term=%22Appel%20RD%22%5BAuthor%5D), [Ou K](http://www.ncbi.nlm.nih.gov/pubmed?term=%22Ou%20K%22%5BAuthor%5D), [Golaz O](http://www.ncbi.nlm.nih.gov/pubmed?term=%22Golaz%20O%22%5BAuthor%5D), [Sanchez JC](http://www.ncbi.nlm.nih.gov/pubmed?term=%22Sanchez%20JC%22%5BAuthor%5D), [Yan JX](http://www.ncbi.nlm.nih.gov/pubmed?term=%22Yan%20JX%22%5BAuthor%5D), [Gooley AA](http://www.ncbi.nlm.nih.gov/pubmed?term=%22Gooley%20AA%22%5BAuthor%5D), [Hughes G](http://www.ncbi.nlm.nih.gov/pubmed?term=%22Hughes%20G%22%5BAuthor%5D), [Humphery-Smith I](http://www.ncbi.nlm.nih.gov/pubmed?term=%22Humphery-Smith%20I%22%5BAuthor%5D), [Williams KL](http://www.ncbi.nlm.nih.gov/pubmed?term=%22Williams%20KL%22%5BAuthor%5D), [Hochstrasser DF](http://www.ncbi.nlm.nih.gov/pubmed?term=%22Hochstrasser%20DF%22%5BAuthor%5D). From proteins to proteomes: large scale protein identification by two-dimensional electrophoresis and amino acid analysis. [Biotechnology (N Y).](http://www.ncbi.nlm.nih.gov/pubmed/9636313##) 1996 Jan;14(1):61-5.
20. [Turtoi A](http://www.ncbi.nlm.nih.gov/pubmed?term=%22Turtoi%20A%22%5BAuthor%5D), [De Pauw E](http://www.ncbi.nlm.nih.gov/pubmed?term=%22De%20Pauw%20E%22%5BAuthor%5D), [Castronovo V](http://www.ncbi.nlm.nih.gov/pubmed?term=%22Castronovo%20V%22%5BAuthor%5D). Innovative proteomics for the discovery of systemically accessible cancer biomarkers suitable for imaging and targeted therapies. [Am J Pathol.](http://www.ncbi.nlm.nih.gov/pubmed/21224037##) 2011 Jan;178(1):12-8.
21. [Trist DG](http://www.ncbi.nlm.nih.gov/pubmed?term=%22Trist%20DG%22%5BAuthor%5D). Scientific process, pharmacology and drug discovery. [Curr Opin Pharmacol.](http://www.ncbi.nlm.nih.gov/pubmed/21704559##) 2011 Oct;11(5):528-33.
22. [Mutch DM](http://www.ncbi.nlm.nih.gov/pubmed?term=%22Mutch%20DM%22%5BAuthor%5D), [Tordjman J](http://www.ncbi.nlm.nih.gov/pubmed?term=%22Tordjman%20J%22%5BAuthor%5D), [Pelloux V](http://www.ncbi.nlm.nih.gov/pubmed?term=%22Pelloux%20V%22%5BAuthor%5D), [Hanczar B](http://www.ncbi.nlm.nih.gov/pubmed?term=%22Hanczar%20B%22%5BAuthor%5D), [Henegar C](http://www.ncbi.nlm.nih.gov/pubmed?term=%22Henegar%20C%22%5BAuthor%5D), [Poitou C](http://www.ncbi.nlm.nih.gov/pubmed?term=%22Poitou%20C%22%5BAuthor%5D), [Veyrie N](http://www.ncbi.nlm.nih.gov/pubmed?term=%22Veyrie%20N%22%5BAuthor%5D), [Zucker JD](http://www.ncbi.nlm.nih.gov/pubmed?term=%22Zucker%20JD%22%5BAuthor%5D), [Clément K](http://www.ncbi.nlm.nih.gov/pubmed?term=%22Cl%C3%A9ment%20K%22%5BAuthor%5D). Needle and surgical biopsy techniques differentially affect adipose tissue gene expression profiles. [Am J Clin Nutr.](http://www.ncbi.nlm.nih.gov/pubmed/19056587##) 2009 Jan;89(1):51-7.

**The effect of vitamin D supplementation on cardiovascular risk factors in subjects with low serum 25-hydroxyvitamin D levels**

Version 5, 221215 (Protocol code number: TromsøEndo-2013-1)

EUDRACT NR: 2013-003514-40. Vitamin D tilskudd og risiko for hjerte-kar sykdom

**Amendment 4, Effects of vitamin D on sleep, version 1, 2016-16-04**

Low serum 25OHD levels are associated with impaired sleep quality and longer time to fall asleep (1, 2). Persistent inadequacy of vitamin D may also increase the risk for obstructive sleep apnea via promotion of adenotonsillar hypertrophy, airway muscle myopathy, and/or chronic rhinitis (3). Furthermore, comparisons of brain regions associated with sleep-wake regulation and vitamin D target neurons in the diencephalon and several brainstem nuclei suggest direct central effects of vitamin D on sleep (4).

However, it is now know whether supplementation with vitamin D improves sleep quality.

We will there in the D-COR study include a questionnaire on sleep pattern and sleep quality at baseline and at the end of the study. The questionnaire will be given to the participants at visit 1 to be filled in at home and returned at visit 2. Similarly, the questionnaire will be given at visit 3 to be filled in at home and returned at visit 4. The questionnaire is the same as that used in the Tromsø study, 7^th^ survey. The format of the questionnaire is such that it will allow optical reading. Amendment 4 will be implemented as soon as it is approved by the Regional Ethics committee and the Clinical Research Unit.

**References**

1. [Çakır T](http://www.ncbi.nlm.nih.gov/pubmed/?term=%C3%87ak%C4%B1r%20T%5BAuthor%5D&cauthor=true&cauthor_uid=25904436),et al. An evaluation of sleep quality and the prevalence of restless leg syndrome in vitamin D deficiency. [Acta Neurol Belg.](http://www.ncbi.nlm.nih.gov/pubmed/25904436) 2015 Dec;115(4):623-7.
2. Shuie I. Low vitamin D levels in adults with longer time to fall asleep: US HANES, 2005-2006. [Int J Cardiol.](http://www.ncbi.nlm.nih.gov/pubmed/23938219) 2013 Oct 12;168(5):5074-5. doi: 10.1016/j.ijcard.2013.07.195. Epub 2013 Jul 27.
3. [McCarty DE](http://www.ncbi.nlm.nih.gov/pubmed/?term=McCarty%20DE%5BAuthor%5D&cauthor=true&cauthor_uid=24075129), [Chesson AL Jr](http://www.ncbi.nlm.nih.gov/pubmed/?term=Chesson%20AL%20Jr%5BAuthor%5D&cauthor=true&cauthor_uid=24075129), [Jain SK](http://www.ncbi.nlm.nih.gov/pubmed/?term=Jain%20SK%5BAuthor%5D&cauthor=true&cauthor_uid=24075129), [Marino AA](http://www.ncbi.nlm.nih.gov/pubmed/?term=Marino%20AA%5BAuthor%5D&cauthor=true&cauthor_uid=24075129). The link between vitamin D metabolism and sleep medicine. [Sleep Med Rev.](http://www.ncbi.nlm.nih.gov/pubmed/24075129) 2014 Aug;18(4):311-9.
4. [Gominak SC](http://www.ncbi.nlm.nih.gov/pubmed/?term=Gominak%20SC%5BAuthor%5D&cauthor=true&cauthor_uid=22583560), [Stumpf WE](http://www.ncbi.nlm.nih.gov/pubmed/?term=Stumpf%20WE%5BAuthor%5D&cauthor=true&cauthor_uid=22583560). The world epidemic of sleep disorders is linked to vitamin D deficiency. [Med Hypotheses.](http://www.ncbi.nlm.nih.gov/pubmed/22583560) 2012 Aug;79(2):132-5.

**Løpenummer** ☐☐☐☐ **Intialer** ☐☐☐☐

**Utfylt ved** ☐ visit 1 ☐ visit 3

**Spørsmål om søvn**

**Hvor mange dager per uke?**

(Kryss av i den ruten som passer best)

|  | **Ingen**  **dager** | **1 dag** | **2 dager** | **3 dager** | **4 dager** | **5 dager** | **6 dager** | **7 dager** |
| --- | --- | --- | --- | --- | --- | --- | --- | --- |
|  |  |  |  |  |  |  |  |  |
| 1. Bruker du mer enn 30 minutter på å sovne inn etter at lysene er slukket? | ☐ | ☐ | ☐ | ☐ | ☐ | ☐ | ☐ | ☐ |
| 2. Er du våken mer enn 30 min innimellom søvnen? | ☐ | ☐ | ☐ | ☐ | ☐ | ☐ | ☐ | ☐ |
| 3. Våkner du mer enn 30 min tidligere enn du ønsker uten å få sove  igjen? | ☐ | ☐ | ☐ | ☐ | ☐ | ☐ | ☐ | ☐ |
| 4. Føler du deg for lite uthvilt etter å ha sovet? | ☐ | ☐ | ☐ | ☐ | ☐ | ☐ | ☐ | ☐ |
| 5. Er du så søvnig/trett at det går utover skole/jobb eller privattid? | ☐ | ☐ | ☐ | ☐ | ☐ | ☐ | ☐ | ☐ |
| 6. Er du misfornøyd med søvnen din? | ☐ | ☐ | ☐ | ☐ | ☐ | ☐ | ☐ | ☐ |

**7. Om du har søvnplager, hvor lenge har de vart?**

☐ Mindre enn en uke

☐ 1-3 uker

☐ 1 måned

☐ 2 måneder

☐ 3 måneder

☐ 4-6 måneder

☐ 7-12 måneder

☐ mer enn 1 år

☐ Har ikke søvnplager

**8. Har du vanligvis skiftarbeid**

☐ Nei

☐ Ja

**9. Når pleier du vanligvis å legge deg for å sove**

| **På arbeidsdager/hverdager** | **På fridager/helgedager** |
| --- | --- |
|  |  |
| ☐ Før 20:00 | ☐ Før 20:00 |
| ☐ 20:00 | ☐ 20:00 |
| ☐ 20:30 | ☐ 20:30 |
| ☐ 21:00 | ☐ 21:00 |
| ☐ 21:30 | ☐ 21:30 |
| ☐ 22:00 | ☐ 22:00 |
| ☐ 22:30 | ☐ 22:30 |
| ☐ 23:00 | ☐ 23:00 |
| ☐ 23:30 | ☐ 23:30 |
| ☐ 00:00 | ☐ 00:00 |
| ☐ 00:30 | ☐ 00:30 |
| ☐ 01:00 | ☐ 01:00 |
| ☐ 01:30 | ☐ 01:30 |
| ☐ 02:00 | ☐ 02:00 |
| ☐ Etter 02:00 | ☐ Etter 02:00 |

**10. Hvor lenge ligger du våken før du sovner**

| **På arbeidsdager/hverdager** | **På fridager/helgedager** |
| --- | --- |
|  |  |
| ☐ Sovner med en gang | ☐ Sovner med en gang |
| ☐ Etter 5 minutter | ☐ Etter 5 minutter |
| ☐ Etter 10 minutter | ☐ Etter 10 minutter |
| ☐ Etter 15 minutter | ☐ Etter 15 minutter |
| ☐ Etter 20 minutter | ☐ Etter 20 minutter |
| ☐ Etter 25 minutter | ☐ Etter 25 minutter |
| ☐ Etter 30 minutter | ☐ Etter 30 minutter |
| ☐ Etter 30 - 45 minutter | ☐ Etter 30 - 45 minutter |
| ☐ Etter 45 - 60 minutter | ☐ Etter 45 - 60 minutter |
| ☐ Etter mer enn 60 minutter | ☐ Etter mer enn 60 minutter |

**11. Når pleier du vanligvis å våkne**

| **På arbeidsdager/hverdager** | **På fridager/helgedager** |
| --- | --- |
|  |  |
| ☐ Før 05:00 | ☐ Før 05:00 |
| ☐ 05:00 | ☐ 05:00 |
| ☐ 05:30 | ☐ 05:30 |
| ☐ 06:00 | ☐ 06:00 |
| ☐ 06:30 | ☐ 06:30 |
| ☐ 07:00 | ☐ 07:00 |
| ☐ 07:30 | ☐ 07:30 |
| ☐ 08:00 | ☐ 08:00 |
| ☐ 08:30 | ☐ 08:30 |
| ☐ 09:00 | ☐ 09:00 |
| ☐ 09:30 | ☐ 09:30 |
| ☐ 10:00 | ☐ 10:00 |
| ☐ 10:30 | ☐ 10:30 |
| ☐ 11:00 | ☐ 11:00 |
| ☐ Etter 11:00 | ☐ Etter 11:00 |

**12. Hvor ofte tar du deg en lur på dagtid**

☐ Aldri, eller sjeldnere enn en gang i måneden

☐ Sjeldnere enn en gang i uka

☐ 1-2 dager i uka

☐ 3-5 dager i uka

☐ Hver dag eller nesten hver dag

**13. Hvis du tar deg en lur, hvor lenge pleier den vanligvis å vare**

☐ 5 minutter

☐ 5 – 15 minutter

☐ 15 – 30 minutter

☐ 30 - 45 minutter

☐ 45 – 60 minutter

☐ 1 time – 1 ½ time

☐ 1 ½ time – 2 timer

☐ 2 – 2 ½ time

☐ 2 ½ - 3 timer

☐ mer enn 3 timer

**14. Snorker du når du sover**

☐ Aldri, eller sjeldnere enn en natt i måneden

☐ Sjeldnere enn en natt i uka

☐ 1-2 netter i uka

☐ 3-5 netter i uka

☐ Hver natt eller nesten hver natt

☐ Vet ikke

**15. Har du opplevd pustestopp når du sover**

☐ Aldri, eller sjeldnere enn en natt i måneden

☐ Sjeldnere enn en natt i uka

☐ 1-2 netter i uka

☐ 3-5 netter i uka

☐ Hver natt eller nesten hver natt

☐ Vet ikke

**16. Hvor sannsynlig er det at du døser av eller sovner i følgende situasjoner**

|  | **Ville aldri døse eller sovne** | **Liten sjanse for å døse eller sovne** | **Moderat**  **sjanse for å døse eller sovne** | **Stor sjanse for å døse eller sovne** |
| --- | --- | --- | --- | --- |
|  |  |  |  |  |
| Sitter og leser | ☐ | ☐ | ☐ | ☐ |
| Ser på TV | ☐ | ☐ | ☐ | ☐ |
| Sitter inaktiv på et offentlig sted (f.eks teater eller møte) | ☐ | ☐ | ☐ | ☐ |
| Legger deg for å hvile om ettermiddagen hvis omstendighetene tillater det | ☐ | ☐ | ☐ | ☐ |
| Sitter og snakker med noen | ☐ | ☐ | ☐ | ☐ |
| Sitter stille etter lunsj (uten å ha inntatt alkohol) | ☐ | ☐ | ☐ | ☐ |
| I en bil som har stoppet none få minutter i trafikken | ☐ | ☐ | ☐ | ☐ |

**17. Bruker du noen form for sovemedisin**

☐ Aldri

☐ Sjeldnere enn en gang i måneden

☐ Sjeldnere enn en gang i uka

☐ 1-2 ganger i uka

☐ 3-5 ganger i uka

☐ Hver eneste dag i uke eller nesten hver dag i uka

**18. Dersom du bruker sovemedisin hva heter den**

………………………………………………….
